# Homocysteine in Neurology: A Possible Contributing Factor to Small Vessel Disease

**DOI:** 10.3390/ijms22042051

**Published:** 2021-02-19

**Authors:** Rita Moretti, Mauro Giuffré, Paola Caruso, Silvia Gazzin, Claudio Tiribelli

**Affiliations:** 1 Department of Medical, Surgical and Health Sciences, University of Trieste, 34149 Trieste, Italy; gff.mauro@gmail.com (M.G.); paolacaruso83@gmail.com (P.C.); 2 Italian Liver Foundation, AREA SCIENCE PARK, 34149 Trieste, Italy; silvia.gazzin@fegato.it (S.G.); ctliver@fegato.it (C.T.)

**Keywords:** homocysteine, SVD, neurodegeneration, neuroinflammation, oxidative stress

## Abstract

Homocysteine (Hcy) is a sulfur-containing amino acid generated during methionine metabolism, accumulation of which may be caused by genetic defects or the deficit of vitamin B12 and folate. A serum level greater than 15 micro-mols/L is defined as hyperhomocysteinemia (HHcy). Hcy has many roles, the most important being the active participation in the transmethylation reactions, fundamental for the brain. Many studies focused on the role of homocysteine accumulation in vascular or degenerative neurological diseases, but the results are still undefined. More is known in cardiovascular disease. HHcy is a determinant for the development and progression of inflammation, atherosclerotic plaque formation, endothelium, arteriolar damage, smooth muscle cell proliferation, and altered-oxidative stress response. Conversely, few studies focused on the relationship between HHcy and small vessel disease (SVD), despite the evidence that mice with HHcy showed a significant end-feet disruption of astrocytes with a diffuse SVD. A severe reduction of vascular aquaporin-4-water channels, lower levels of high-functioning potassium channels, and higher metalloproteinases are also observed. HHcy modulates the N-homocysteinylation process, promoting a pro-coagulative state and damage of the cellular protein integrity. This altered process could be directly involved in the altered endothelium activation, typical of SVD and protein quality, inhibiting the ubiquitin-proteasome system control. HHcy also promotes a constant enhancement of microglia activation, inducing the sustained pro-inflammatory status observed in SVD. This review article addresses the possible role of HHcy in small-vessel disease and understands its pathogenic impact.

## 1. Introduction

**Figure 1:**
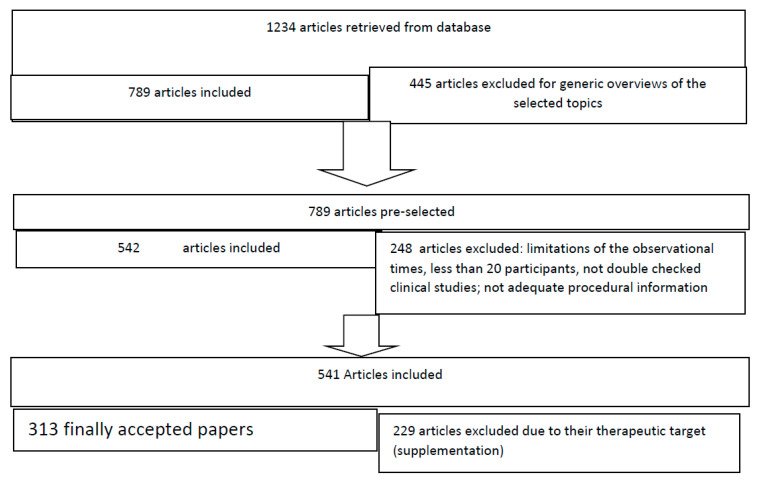
Flowchart of search strategy and selection criteria.

This article aims to define the role of Hcy in the development of small vessel disease (SVD) and neurological damage. We searched MEDLINE using the search terms “vascular dementia,” “subcortical vascular dementia,” “vascular cognitive impairment,” “small vessel disease,” “arteriolosclerosis,” “cerebral flow regulation,” “homocysteine” “neurovascular coupling,” “endothelium,” smooth muscle cells arteries”, “neuroinflammation” “oxidative damage”, and “neurodegeneration”. Publications were selected mostly from the past 25 years (1 January 1995 to 15 December 2020) but did not exclude frequently referenced and highly regarded older publications. The research has been extended with the same strings to EMBASE, COCHRANE LIBRARY, and LILACS databases. We have considered papers published in English, French, German, and Italian. Secondary searching was performed using the most relevant articles (following PRISMA statement, 2009) [1]. Congress abstracts and isolated case reports were not considered. We (all the Authors who contributed to the research strategy) searched the reference lists of articles identified by this search strategy and selected those we judged relevant. Review articles and book chapters are cited for providing additional details. A total of 1234 studies showed up, and appropriate studies (*n* = 312) were included. The authors carefully read all the eligible articles (Figure 1).

## 2. Cerebral Small Vessel Disease

Cerebral small vessel disease (SVD) primarily distresses the small perforating arteries, defined as vessels with less than 50 μm diameters, which perfuses the deep brain structures, the meningeal space, and the white substance [2,3]. SVD progression leads to the condition known as subcortical vascular dementia (sVAD), one of the most common forms of degenerative disorders globally, accounting for 45% of dementia cases in the world [4,5,6]. While affecting the small arteries, SVD contributes to enlargement and a loss of function of perivascular spaces (PVS) [7,8], critical in catabolic/glynphatic responses [9,10,11,12,13], as well as occlusion of small draining veins [14], with a disruption of the blood–brain barrier (BBB) [4]. The sum of all the events promotes a chronic inflammatory status, which is the pathological basis of SVD [12,15,16,17]. Small arteries undergo a pathological process named arteriolosclerosis [4,9,18,19,20], which primarily impedes the autoregulation of cerebral blood flow (CBF) exerted by small arteries [9,21,22,23,24]. Arteriolosclerosis occurs in two primary histological forms: hyperplastic and hyaline [25,26,27,28]. A reduction of the arterial elasticity, a loss of control of the resting flow, and decreased perfusion pressure towards the profound arteries are observed [29,30,31,32,33,34,35].

In the animal SVD models, there is a reduction of vasopressin and histamine, a direct consequence of the progressive disruption of neural tracts, extending from the supra-optic and tuberomammillary nuclei to the basal forebrain [9,36,37,38]. A super-imposed endothelium-mediated altered baroreflex activity is associated with low-level functions of the autonomic nervous system [39,40,41,42]. The main consequence is a decline of the cerebral blood flow control, altering the retrograde vasodilatation system [9,43,44,45,46,47,48,49,50,51,52,53,54,55,56,57,58,59,60,61,62,63]. The chronic hypoperfusion condition promotes a chronic inflammatory status, induced by glynphatic, veins, and BBB disruption. 

In SVD, there is a severe oligodendrocyte degeneration, a microglial activation (testified by a severe increase of caspase 3-RNA and matrix-metalloprotease 2 (MMP-2) expression), massive calcium inflows, and apoptosis process [34,35]. Astrocytes respond in a two-way system: in the first ischemic period, they proliferate [35,58], but when the neuroinflammation endures, they lose their end-feet, degenerate, and rapidly die [35,57,58,59,60]. Astrocytic death corresponds to an altered neurovascular coupling and a consequent induction of neuronal death [4,9,64,65,66,67,68,69,70,71]. 

The endothelium is indirectly affected by the apparent mitochondrial damage [72,73], mainly due to a loss of response to the main endothelium-derived nitric oxide-vasodilators [74], prostacyclin [75], and endothelium-derived hyperpolarizing factors (EDHF) [76,77,78,79,80,81]. The general neuroinflammation present in SVD determines a hyperproduction of peroxynitrite [82,83]. It depends on an altered redox response associated with a reduction of the activity of endothelial NO synthase (eNOS) and downregulation of the Rho-associated protein kinase (ROCK), which usually promotes the vascular endothelium growth factor (VEGF) in response to vascular injury [84,85,86,87,88,89,90,91,92,93]. 

NO is a significant mediator of vasodilatation through cGMP/PKG signals, leading to decreased Ca2+ concentration. Besides, NO-mediated signals trigger an increase in myosin light-chain phosphatase (MLCP) activity. ROCK inactivates MLCP via calcium desensitization [84,94] and therefore decreases the availability of NO [95,96]. 

Altered endothelium activation is not specific to gray matter [71,97,98,99], but is more pronounced in white matter, putamen, caudate, and in all the basal forebrain-frontal subcortical networks [100,101,102,103,104,105,106,107,108,109]. 

**Figure 2:**
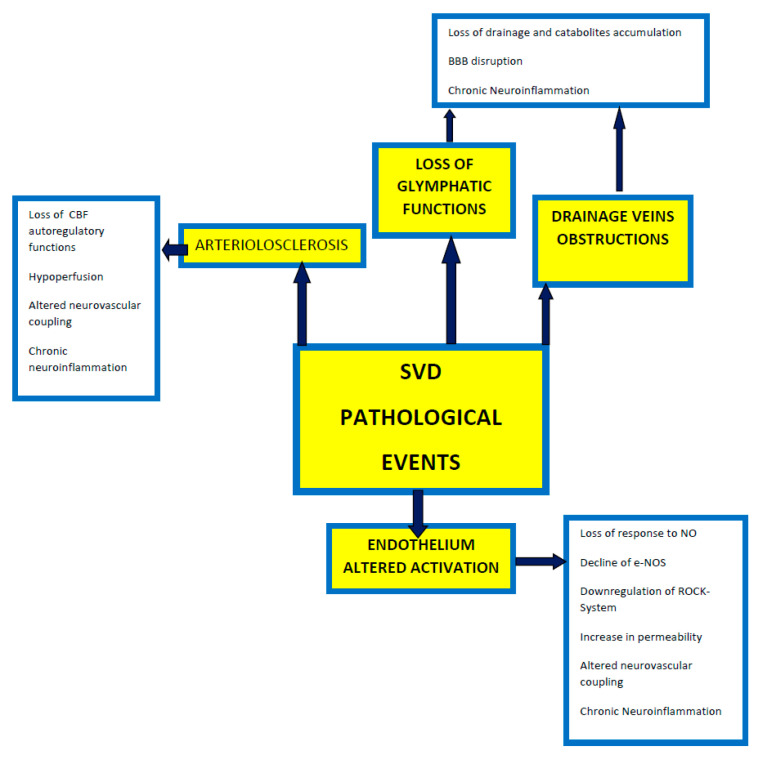
A synoptical overview of the pathological events which occur in SVD (Small Vessel Disease) (abbreviations: BBB = blood–brain barrier; eNOS = endothelium-derived NO synthase; NO = Nitric oxide; ROCK = Rho-associated protein kinase; SVD = Small Vessel Disease).

Taken together, SVD is a progressive disease [3,8,38,110,111,112,113,114,115,116,117,118,119], though the exact timing of its progression is not established [9]. The rapid confluence of the isolated white matter lesions, the number of silent infarcts, and the vascular lacunar events are essential in determining the cognitive and behavior impairment during SVD [4,9,38,117,118,119,120,121,122,123,124,125,126,127,128,129,130,131] (Figure 2).

## 3. Homocysteine and Brain 

Hcy is a sulfur-containing intermediary amino acid [132], recycled via the remethylation pathway or converted into cysteine via the trans-sulfuration pathway [4]. 

The methionine synthesis occurs when there is a reduction of 5,10-methylenetetrahydrofolate to 5-methyltetrahydrofolate (5-methylTHF) [133,134,135]. In remethylation, Hcy acquires a methyl group from N-5-methyltetrahydrofolate or from betaine to form methionine. The reaction with N-5-methyltetrahydrofolate occurs in all tissues and is vitamin B12-dependent. In particular, methionine adenosyltransferase (MAT) catalyzes S-adenosylmethionine (AdoMet) (SAM), actively consuming Adenosyn triphosphate (ATP) [133,134]. SAM is the methyl group donor in numerous methylation reactions, a fundamental process for the protein, phospholipid, and biogenic amines synthesis [136,137,138,139,140,141]. Every reaction made by methyltransferases produces S-adenosylhomocysteine (AdoHcy) (SAH) [142,143,144,145]. The SAM to SAH ratio defines the cell’s methylation potential [146,147,148,149,150,151,152,153,154,155]. 

In the trans-sulfuration pathway, Hcy condenses with serine to form cystathionine. It is an irreversible reaction catalyzed by the pyridoxal-50-phosphate (PLP)-containing enzyme, cystathionine β-synthase. Cystathionine is hydrolyzed by a second PLP-containing enzyme, γ-cystathionase, to form cysteine and α-ketobutyrate [144]. Excess cysteine is oxidized to taurine or inorganic sulfates or is excreted in the urine [144]. Therefore, the trans-sulfuration pathway catabolizes excess homocysteine, which is not required for methyl transfer [144,151,152,153,154,155]. 

The intrinsic capacity to differentiate between the remethylation and trans-sulfuration pathways to adapt to different intake-methionine levels in the diet strongly implies the existence of a coordinate regulation between these two pathways [144]. SAM could act as an allosteric inhibitor of methylenetetrahydrofolate reductase (MTHFR). It could also play a role as an activator of cystathionine β-synthase, promoting the trans-sulfuration pathway (cystathionine synthesis) [144]. When the methionine supply is low, there is an elevated rate of N-5-methyltetrahydrofolate production. Thus, remethylation will be favored over trans-sulfuration because the concentration of SAM is too low to activate the cystathionine β-synthase enzyme [144]. Remethylation of Hcy to methionine (the methionine cycle) predominates over the catabolic degradation of Hcy (trans-sulfuration) because of the order of magnitude difference in Km between MS and CBS [155,156,157,158,159,160]. 

The methylation reactions are necessary for the brain, SAM being the sole methyl group donor in numerous methylation reactions involving proteins, phospholipids, and biogenic amines, and packaging many phospholipids, i.e., polyunsaturated phosphatidylcholines (PC). 

Hyper-homocysteinemia (HHcy) is defined as levels > 15 mol/L, levels between 15 and 30 are considered moderate HHcy, levels at 30–100 micro-mol/L are considered intermediate/severe HHCy, and levels above 100 micro-mol/L are considered as severe (often fatal) HHcy [156,157]. Hcy levels are inversely related to food supplements, principally folate and vitamin B12 [158,159,160], and directly related to smoking, alcohol, physical apathy [161,162], and aging [163]. In vitro studies that explored the correlation between Hcy and inflammation, neurodegeneration, atherosclerosis, and oxidative damage have been inconclusive. Similarly, in vivo trials failed to demonstrate a real benefit in clinical conditions when Hcy is abated by vitamin B12 or B9 supplementation [164].

Genetic causes of severe HHcy linked to a deficiency of CBS or other alterations of remethylation and trans-sulfuration pathways have been reported in neural tube defects and blood–brain barrier alterations [165,166,167,168,169]. Clinical and experimental works demonstrate that HHcy decreases the cell’s methylation potential, modifying the SAM/SAH ratio [170,171,172], and this is the primary determinant for a generalized DNA hypomethylation associated with an excess of oxidative stress [144,170].

**Figure 3:**
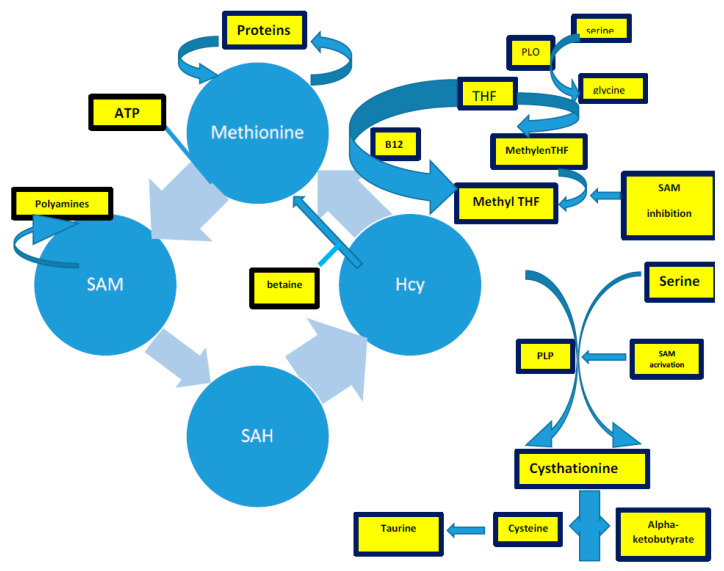
The complex of Hcy production, described in the text. Acronyms: SAM: s-adenosylmethionine; THF: tetrahydrofolate; PLP: pyridoxal-5-phosphate.

Homocysteine accumulation could interfere with endothelium dysregulation, favor oxidative damage, and promote neuroinflammation and neurodegenerative processes [163,171,172,173,174,175]. All these processes occur in SVD; nevertheless, few studies directly focus on HHcy and SVD. Our review attempts to shed some light on the three principal mechanisms of HHcy-induced damage, trying to focus on SVD (Figure 3).

## 4. Homocysteine and Neurodegeneration

HHcy is linked to neurodegeneration, starting from the well-known relationship between its elevation during aging. Many in vivo and in vitro studies showed that HHcy favors the Abeta1–40 deposition in AD [174], mediated by an Hcy-induced upregulation of the Endoplasmic Reticulum Protein (HERP). HERP favors the c-secretase enzyme activity and the consequent increment of the intra- and extra-cellular accumulation of Abeta1–40 and Abeta 42 [175,176,177,178]. 

Hcy is strongly related to neurodegenerative/neuroinflammation conditions by the homocysteinylation process. Homocysteinylation leads to protein damage, i.e., protein denaturation, enzyme inactivation, inflammatory activities, and amyloid-oligomers deposition [179,180,181,182,183,184,185]. Under normal metabolic conditions, the cellular synthesis of Hcy thiolactone is rather low because intracellular concentrations of Hcy are relatively low [186]. If Hcy levels are increased because of a reduction in transmethylation and/or trans-sulfuration, Hcy thiolactone synthesis is enhanced—it could be as much as 60% of the metabolized Hcy [186]. Hcy can be linked to a protein via an isopeptide bond to lysine (Lys) residues (N-Hcy-protein) [187,188,189] or via a disulfide bond to Cys residues (S-Hcy-protein) [190,191,192,193]. N-homocysteinylation is an emerging post-translational protein modification that impairs or alters the protein’s structure/function and causes protein damage [194]. There are two limiting processes of the N-homocysteinylation: the quantity of cyclic Hcy-thiolactone (dependent on HHcy) and the number of lysine residues encountered [195,196,197]. The most evident result of the general homocysteinylation process is protein aggregation and virtual protein misfolding. Thus, Hcy-thiolactone induces apoptosis directly in endothelial cell cultures in in vitro and in vivo models [195]. 

Hcy is also linked to neurodegenerative pathology by influencing tau phosphorylation. As previously described [4,9], tau protein has many functions: the correct assembly of microtubules, directing, therefore, the axonal micronutrients transport toward the neuronal soma. The active form of tau needs constant dephosphorylation mediated by methyltransferase systems (the so-called PPM1 and PPM2A), and the methylation occurs through SAM-dependent reactions [198,199,200,201,202]. Tau hyperphosphorylation has two direct consequences: (1) the disaggregation of microtubules, which leads to an inhibition of axonal transport, and (2) a neuronal death, together with a deposition of damaged microtubules, which forms the so-called tau depositions, or neurofibrillary tangles [203,204,205]. These phenomena have always been associated with degenerative conditions (AD, frontal Pick complex, etc.), but they have also been demonstrated in neuroblastoma cultured cells when the culture medium is depleted by folate, and an increase of P-tau by 66% occurs [206].

HHcy has an intrinsic toxic property [4,9,207] as it acts as an agonist of NMDA (N-methyl D-Aspartate) receptors [208,209,210,211] depending on glycine concentration. Hcy acts as a partial antagonist of the NMDA receptors [4,162,171,207,208], but when the glycine concentration is increased (like in the brain ischemia, in vasospasms, i.e., in prolonged migraine aura attack), even low doses of Hcy could act as an agonist of NMDA channels [212,213], inducing an enhancement of calcium flows [213]. HHCy promotes an extracellular signal-regulated kinase activity in the hippocampus, regulated or blocked by three glutamate receptor antagonists (NMDA, not-NMDA, and metabotropic receptors) [154,214]. It has been suggested that Hcy could directly activate group I metabotropic glutamate receptors, favoring calcium influx currents [212].

Collectively, HHcy exerts essential alteration in the SVD pattern. HHcy induces an increase of Abeta 1–40 toxicity on the smooth muscle cells of the brain’s small arteries, where cerebral amyloid depositions occur, transforming the event into cerebral amyloid angiopathy (CAA), a constant finding in overt SVD condition [4,9,215,216,217]. Moreover, the HHcy condition enhances the m-RNA (Messanger-RNA) production of the C-reactive protein (CRP), over-expressing the NR1 subunit of NMDA receptor expression [4,218]. HHcy enhances the signal pathway cascade, mediated by CRP hyperproduction, mediated by NMDA-ROS-erk1/2/p38-NFK-Beta (NFK = Nuclear Kappa Factor-Beta), which occurs in the smooth muscle cells’ brain small arteries [218]. Homocysteinilation promotes apoptosis [195], endothelium alterations, protein misfolding, and protein aggregation. In fact, the multiple lysine-rich proteins are fibrinogen [196,219], high-density lipoprotein [220], lysine oxidase [221], and cytochrome c [197], and all of them homocysteinylate, aggregate [195], and lead to a general pro-thrombotic condition [196,220,221,222], enhanced coagulation [223], and reduced fibrinolysis [224,225].

## 5. Homocysteine and Neuroinflammation

The pivotal role of HHcy in neuroinflammation is the acceleration of the lipid peroxidation derived from the disruption of the redox system in vascular endothelium, and consequently, among neural cells [226,227,228]. HHcy is always present in multiple traumatic damages, sepsis, multi-organic failure, etc., and HHCy is a sign of poor prognosis [229,230]. 

Animal models showed that HHcy promotes the increase of TNF-alpha, IL1-beta, is inversely associated with a diminution of cystathionine-gamma-lyase-derived H2S in macrophages, and upregulates the transcriptional fibroblast growth factor-2 [9,231,232,233,234]. HHcy directly acts on the endothelium by inducing an upregulation of IL-6, IL-8, TNF-alpha expression [235,236,237], together with cathepsins, involved in the endothelium-inflammatory and vascular remodeling processes [238,239], by influencing IL-6 and TNF-alpha [240,241,242,243,244,245] and enhancing the VEGF/ERK1/2 signaling pathway [240,241,242,243,244,245,246,247], which is a constant in the atherosclerosis process [247]. 

HHCy plays like an agonist of NMDA receptors in CNS (Central Nervous System) and neutrophils and macrophages whenever glycine increases [213]. HHcy activates NMDA receptors, inducing a significant intra-cytoplasmic calcium inflow, with the consequent lipoperoxidation inflammatory process, hyper-activation of the oxidative process accumulation of ROS species [248,249,250]. HHcy also induces a pro-inflammatory status by direct interference with B-control. An in-vitro study demonstrated that there is an upregulation of pyruvate kinase muscle isoenzyme 2 (PKM-2), B-mediated, inhibited by shikonin [251], which mainly promotes the inflammatory basis of atherosclerosis cascade [162,171,251].

HHcy is correlated to a higher quantity of asymmetric dimethylarginine (ADMA), which acts as an inhibitor of eNOS, which catalyzes the production of NO from arginine [252,253,254,255,256]. Together with elevated levels of ADMA, HHcy promotes an increase of the endoplasmic reticulum (ER) stress, upregulating metalloproteinases-9 (MMP-9), and inducing apoptosis [244,255,256,257,258,259,260].

A very new light has been shed on the endothelium effects of HHcy, mediated by ER stress and unfolded protein response (UPR), both events promoting apoptosis in endothelial cells [261,262]. UPR usually upregulates the ER and promotes increased chaperon production, controlling the transcription and translation process, and downregulating the ER proteins [262,263,264,265]. Thus, when there is a hyper-induction of ER stress induced by HHcy [195], there is an accumulation of protein folding capacity. The overwhelming protein accumulation promotes cell modifications, alterations of cell pseudopods, loss of cell adhesion capacity, and caspase-mediated cell death [264,265].

Attention has recently been dedicated to the pro-inflammatory effect of HHcy, exerted directly on smooth muscle cells: HHCy mouse models were found to have enhanced expression of the receptors for the AGEs or vascular cell adhesion molecule [222,223], and MMP-9 [196]. The inflammation cascade could be mediated by the effects on smooth muscle cells rather than on the endothelium alterations [194,264,266,267,268]. The effect of Hcy on B and T cells’ modulation is still undefined, although recent in vitro data suggest B lymphocytes’ activation.

HHcy exerts an overt effect on the global cellular protein quality control (PQC), essential for proteome integrity and cell viability [269]. HHcy has been demonstrated to reduce chaperone levels and impair the UPR systems and control process [269,270,271]. HHcy mice models enhance brain microglia by expressing pro-inflammatory cytokines [272,273,274,275,276,277,278,279,280], particularly the signal transducer and activator of transcription3 (the so-called STAT3). STAT3 helps the microglial regulation of different pro-inflammatory genes [278], such as Il-1-beta, TNF-alpha, and Il-6 [279,280].

## 6. Homocysteine and Oxidative Stress

Oxidative damage is the most accepted consequence of HHcy [9,162,171,220,281,282,283,284,285] and is linked to the oxidation process of the free thiol group of Hcy when it binds many different proteins, such as albumin, other low-weight plasma thiols, or other molecules of Hcy.

Four different mechanisms have been proposed to explain the oxidative stress induced by HHcy [227], not self-excluding: (1) a possible auto-oxidation induced by Hcy, (2) general inhibition of the cellular antioxidant enzymes, (3) NOS-derived production of superoxide anion, through a direct uncoupling of the eNOS, and the disruption of the extracellular superoxide dismutase of the endothelium, and (4) via direct activation of NADPH oxidases [227], that seems to occur directly in the microglia, inducing a hyperactivation of it [286,287]. It has been well-documented that excessive activation of NADPH oxidases contributes to the pathogenesis of numerous peripheral inflammation-related diseases, such as atherosclerosis, diabetes, hypertension, ischemic stroke, and cardiovascular diseases. As a significant superoxide-producing enzyme complex, phagocytic NADPH oxidase (PHOX) is essential for host defense. The discovery of PHOX and non-phagocytic NADPH oxidases in astroglia and neurons further reinforces NADPH oxidases’ critical role in oxidative stress-mediated chronic neurodegeneration [286]. Physiologically, NADPH oxidase-derived ROS have been implicated in the regulation of vascular tone by modulating vasodilation directly (H2O2 may have vasodilator actions) or indirectly by decreasing NO bioavailability (mediated by ·O2− to form ONOO−) [287]. ROS is involved in inflammation, endothelial dysfunction, cell proliferation, migration and activation, fibrosis, angiogenesis, cardiovascular remodeling, and atherosclerosis. These effects are mediated through redox-sensitive regulation of multiple signaling molecules and second messengers, including mitogen-activated protein kinases, protein tyrosine phosphatases, tyrosine kinases, proinflammatory genes, ion channels, and Ca2+ [287].

**Figure 4:**
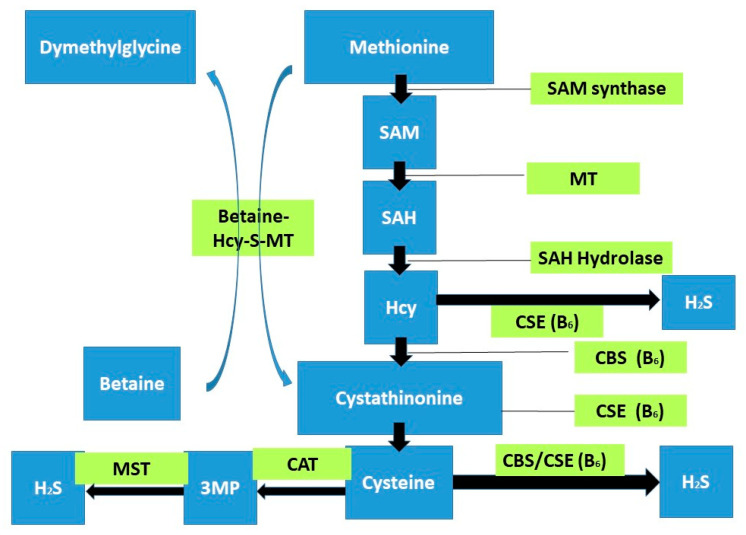
A schematic overview of the association between homocysteine and H2S is represented. Homocysteine is biosynthesized from methionine by S-adenosyl methionine (SAM) synthetase, methyltransferase (MT), and s-adenosyl-L-homocysteine (SAH) hydrolase. Hcy can be either remethylated to methionine (see Figure 2) or trans-sulfurated to cysteine under the catalysis of cystathionine beta-synthase (CBS) and cystathionine gamma-lyase (CSE) that requires vitamin B6 as a cofactor. Hcy and cysteine are substrates for H2S production, and the generation of H2S is catalyzed by CBS, CSE, and 3-mercaptopyruvate sulfurtransferase (MST).

Trans-sulfuration of homocysteine, catalyzed by the vitamin B6-dependent enzymes, produces cystathionine β-synthase (CBS) and cystathionine γ-lyase (CSE). CBS converts homocysteine and serine into cystathionine, which CSE takes up to generate cysteine. CBS and CSE are also the major enzymes responsible for the biogenesis of hydrogen sulfide (H2S), a gasotransmitter known for its regulatory role in many physiological processes. HHcy causes a decrease in H2S production in mice models, the hippocampus [288], and the cardiovascular system, reducing its cardio-protective effects [288] (Figure 4).

HCy can cause endothelial damage by the effect of lectin-like oxidized low-density lipoprotein receptor-1 (LOX-1) DNA methylation through toll-like receptor 4 (TLR4)/nuclear factor (NF)-κB/DNA methyltransferase (DNMT1) [289], allowing ox-LDL (Oxidized Low-Density Lipoproteins) to accumulate in the sub-endothelial layer and promoting atherosclerotic plaques’ formation [289,290,291,292]. These reactions promote pro-coagulative status (directly mediated by platelet activation and through the N-homocysteinylation of fibrinogen and other pro-coagulative proteins [220,221,222]). Moreover, HHcy (directly and by the HHcy-mediated inhibition of Dimethylarginine dimethylaminohydrolase (DDAH), which causes an ADMA accumulation), induced ROS production decreases NO production and bioavailability, triggering increased redox signaling [293,294,295,296,297].

ROS accumulation’s oxidative stress is the primary mechanism that mediates homocysteine-induced vascular injury in SVD and endothelium dysregulation [298,299,300,301,302]. A study on neuroblastoma cells incubated with HHcy [303] determined different time- and concentration-dependent results [4,162,171,303]. This study suggests the potential genotoxic stress, time-exposure, and Hcy concentration relationship on endothelial and smooth muscle cells [303]. At the very beginning, HHcy induces a correct endothelium response, mediated by the formation of S-nitrose-Hcy, which is an endothelium protector factor [303]. Longer HHcy exposure induced a downregulation of eNOS and provoked oxidative damages [293,304,305,306,307,308,309,310,311,312,313].

## 7. Conclusions

The interplay between HHcy and SVD is relatively novel. Only a few studies have been written during the last six months, defining a potential role of homocysteine inside the complexity of SVD pathogenesis [314,315,316]. A very recent study showed a dose-independent relationship between the plasma Hcy levels and the development of SVD [317]. The study needs to be confirmed in a much larger number of patients. Moreover, a relatively recent study produced contradictory results in coronary stenosis, the prevalence of significant coronary artery stenosis, atherosclerotic, calcified, mixed, and non-calcified plaques increased with homocysteine. However, after adjusting cardiovascular risk factors, there were no statistically significant differences in the adjusted odds ratios for atherosclerotic plaque and mixed plaques between the third and first homocysteine tertiles. In asymptomatic individuals, homocysteine is not associated with an increased risk of subclinical coronary atherosclerosis [318]. These results need better and dedicated new works. While many studies focused on thrombosis and HHcy, HHcy and coronary disease, stroke, and major vessel disease, few data are available on HHcy and vascular and neurodegeneration because SVD in the brain is a relatively recent entity. SVD is a complex clinical entity linked to the aging modification of the small arteries, altered endothelium activation, oxidative damage, and generally by a chronic inflammatory state induced by persistent hypoperfusion. Inflammation, oxidative damages, misfolding, and neurodegeneration happen together in a dynamic sequence during the development of SVD. Hcy’s role could change in the temporal sequences of events. Definition of the different roles of Hcy at the different cellular levels, promotion of the confluency of altered white matter areas, and times of the development of SVD in the brain may provide hints as to the modulation of Hcy to prevent disease.

## Data Availability

The data presented in this study are openly available in all the articles cited in references. Data sharing not applicable.

## Abbreviations

5-methylTHF	5:10-methylenetetrahydrofolate to 5-methyltetrahydrofolate
ATP	Adenosine triphosphate
SAHH	AdoHcy hydrolase
AGEs	Advanced glycation end products
AD	Alzheimer’s disease
APP	Amyloid precursor
ADMA	Asymmetric dimethylarginine
BHMT	Betaine-homocysteine methyltransferase
BBB	Blood–brain barrier
CAA	Cerebral amyloid angiopathy
CBF	Cerebral blood flow
SVD	Cerebral small vessel disease
CSF	Cerebrospinal fluid
CRP	C-reactive protein
cGMP	Cyclic guanosine monophosphate
cGMP	Cyclic guanosine-30,5-monophosphate
CBS	Cystathionine β-synthase
CSE	Cystathionine γ-lyase
DDAH	Dimethylarginine dimethylaminohydrolase
DUSPs	Dual-specificity phosphatases
HERP	Endoplasmic protein
ER	Endoplasmic reticulum
eNOS	Endothelial NO synthase
EDHF	Endothelium-derived hyperpolarizing factors
ERK	Extracellular signal-regulated kinase
ERM	Ezrin, radixin, and moesin
GABA	Gamma-amino butyric
PQC	Global cellular protein quality control
Hcy	Homocysteine
HERP	Hcy-induced Endoplasmic Reticulum Protein
HHcy	hyperhomocysteinemia
P-tau	Hyperphosphorylated tau protein
HIF 1	Hypoxia-inducible factor 1
ICAM-1	Intercellular adhesion molecule-1
IL-6	Interleukin-6
LOX	Lectin-like low-density lipoprotein
MMP	Matrix-metalloprotease
MST	3-mercaptopyruvate sulfurtransferase
MAT	Methionine adenosyltransferase
MTR	Methionine synthase
MetRS	Methionyl-tRNA synthase
MT	Methyltransferase
PPM1 and PPM2A	Methyltransferase systems
MAP	Mitogen-activated protein kinases
MLCP	Myosin light-chain phosphatase
NADPH	Nicotinamide Adenine Dinucleotide Phosphate Hydrogen
NO	Nitric oxide
NOS	Nitric oxide synthase
NMDA	N-methyl D-Aspartate
SID	N-[(1,1-dimethyl ethoxy)carbonyl]-L-tryptophan-2-[[[2-[(2- ethyl phenyl)amino]-2-oxoethyl]thio]carbonyl]hydrazide
NFK-Beta	Nuclear factor kappa light-chain enhancer of activated B cells
PVS	Perivascular spaces
PAI-1	Plasminogen activator inhibitor-1
PHOX	Phagocytic NADPH oxidase
PC	Phosphatidylcholines
PEMT	Phosphatidylethanolamine N-methyltransferase
PS1	Presenilin 1
PRMTs	Protein arginine N-methyltransferases
PKM-2	Pyruvate kinase muscle isoenzyme 2
ROS	Reactive oxygen species
ROCK	Rho-associated protein kinase
SAM	S-adenosyl methionine
AdoHcy	S-adenosylhomocysteine
SAH	S-adenosyl-L-homocysteine
AdoMet	S-adenosylmethionine
STATs	Signal transducer and activator of transcription proteins
STAT3	Signal transducer and activator of transcription 3
SIRT1-HSF1 axis	Sirtuin/heat shock factor 1/heat shock protein axis
sTM	Soluble thrombomodulin
sVAD	Subcortical vascular dementia
SOD	Superoxide dismutase
DNMT	Toll like receptor/NF-KB-DNA methyltransferase
TLR	Toll-like receptor
UPS	Ubiquitin-proteasome system
UPR	Unfolded protein response
VEGF	Vascular endothelial growth factor
Cbl	Vitamin B12

## References

[1] Liberati A., Altman D.G., Tetzlaff J., Mulrow C., Gotzsche P.C., Ioannidis J.P.A., Clarke M., Devereaux P.J., Kleijnen J., Moher D. (2009). The PRISMA statement for reporting systematic reviews and meta-analyses of studies that evaluate health care interventions: Explanation and elaboration. J. Clin. Epidemiol..

[2] Pantoni L., Gorelick P. (2014). Cerebral Small Vessel Disease.

[3] Xu W.H. (2014). Large artery: An important target for cerebral small vessel diseases. Ann. Transl. Med..

[4] Moretti R., Caruso P. (2019). The Controversial Role of Homocysteine in Neurology: From Labs to Clinical Practice. Int. J. Mol. Sci..

[5] Vinciguerra L., Lanza G., Puglisi V., Fisicaro F., Pennisi M., Bella R., Cantone M. (2020). Update on the Neurobiology of Vascular Cognitive Impairment: From Lab to Clinic. Int. J. Mol. Sci..

[6] Pantoni L. (2010). Cerebral small vessel disease: From pathogenesis and clinical characteristics to therapeutic challenges. Lancet Neurol..

[7] Zhang E.T., Inman C.B., Weller R.O. (1990). Interrelationships of the pia mater and the perivascular (Wirchov-Robin) spaces in the human cerebrum. J. Anatom..

[8] Iadecola C. (2017). The neurovascular Unit coming of age: A journey through neurovascular coupling in health and disease. Neuron.

[9] Caruso P., Signori R., Moretti R. (2019). Small vessel disease to subcortical dementia: A dynamic model, which interfaces aging, cholinergic dysregulation and the neurovascular unit. Vasc. Health Risk Manag..

[10] Sweeney M.D., Sagare A.P., Zlokovic B.V. (2018). Blood-brain barrier breakdown in Alzheimer disease and other neurodegenerative disorders. Nat. Rev. Neurol..

[11] Abbott N.J., Pizzo M.E., Preston J.E., Janigro D., Thorne R.G. (2018). The role of brain barriers in fluid movement in the CNS: Is there a ‘glymphatic’ system?. Acta Neuropathol..

[12] Huijts M., Duits A., Staals J., Kroon A.A., Leeuw P.W.D., Oostenbrugge R.J.V. (2014). Basal ganglia enlarged perivascular spaces are linked to cognitive function in patients with cerebral small vessel disease. Curr. Neurovasc. Res..

[13] Jiménez-Balado J., Riba-Llena I., Garde E., Valor M., Gutiérrez B., Pujadas F., Delgado P. (2018). Prevalence of hippocampal enlarged perivascular spaces in a sample of patients with hypertension and their relation with vascular risk factors and cognitive function. J. Neurol. Neurosurg. Psychiatry.

[14] Garcia J.H., Lassen N.A., Weiller C., Sperling B., Nakagawara J. (1996). Ischemic stroke and incomplete infarction. Stroke.

[15] Dalkara T., Alarcon-Martinez L. (2015). Cerebral micro-vascular signaling in health and disease. Brain Res..

[16] Giannakopoulos P., Gold G., Kowaru E., von Gunten A., Imhof A., Bouras C., Hof P.R. (2007). Assessing the cognitive impact of Alzheimer disease pathology and vascular burden in the aging brain: The Geneva experience. Acta Neuropathol..

[17] Launer L.J., Hughes T.M., White L.R. (2011). Microinfarcts, brain atrophy, and cognitive function: The Honolulu Asia Aging Study Autopsy Study. Ann. Neurol..

[18] Munoz D.G., Hastak S.M., Harper B., Lee D., Hachinski V.C. (1993). Pathologic correlates of increased signals of the centrum ovale on magnetic resonance imaging. Arch. Neurol..

[19] Mirski M.A. (2005). Pharmacology of Blood Pressure Management during Cerebral Ischemia.

[20] Wallin A., Blennow K., Gottfries C.G. (1989). Neurochemical abnormalities in vascular dementia. Dementia.

[21] Jani B.I., Rajkumar C. (2006). Ageing and vascular ageing. Postgrad. Med. J..

[22] De la Torre J.C. (2002). Vascular basis of Alzheimer’s pathogenesis. Ann. N. Y. Acad. Sci..

[23] Mathias C.J., Kimber J.R. (1999). Postural hypotension: Causes, clinical features, investigation, and management. Annu. Rev. Med..

[24] Roriz-Filho J.S., Bernardes Silva Filho S.R., Rosset I., Roriz-Cruz M., Halliday J.T. (2009). Postural blood pressure dysregulation and dementia: Evidence for a vicious circle and implications for neurocardiovascular rehabilitation. Cardiac Rehabilitation.

[25] Kumar V., Cotran R.S., Robbins S.L. (2007). Basic Pathology.

[26] Lodder J., Bamford J.M., Sandercock P.A., Jones L.N., Warlow C.P. (1990). Are hypertension or cardiac embolism likely causes of lacunar infarction?. Stroke.

[27] Gamble C. (1986). The pathogenesis of hyaline arteriosclerosis. Am. J. Pathol..

[28] Moritz A., Oldt M. (1937). Arteriolar sclerosis in hypertensive and non-hypertensive individuals. Am. J. Pathol..

[29] Pavelka M., Roth J. (2010). Hyaline Arteriolosclerosis. Functional Ultrastructure.

[30] Najjar S.S., Scuteri A., Lakatta E.G. (2005). Arterial aging: Is it an immutable cardiovascular risk factor?. Hypertension.

[31] O’Rourke M.F., Safar M.E. (2005). Relationship between aortic stiffening and microvascular disease in brain and kidney: Cause and logic of therapy. Hypertension.

[32] Cervós-Navarro J., Matakas F., Roggendorf W., Christmann U. (1978). The morphology of spastic intracerebral arterioles. Neuropathol Appl. Neurobiol..

[33] Wakita H., Tomimoto H., Akiguchi I., Kimura J. (1994). Glial activation and white matter changes in the rat brain induced by chronic cerebral hypoperfusion: An immunoistochemical study. Acta Neuropathol..

[34] Farkas E., Donka G., de Vous R.A.I., Mihaly A., Bari F., Luiten P.G.M. (2004). Experimental cerebral hypoeprfusion induces white matter injury and microglial activation in the rat brain. Acta Neuropathol..

[35] Zhang Z.G., Bower L., Zhang R.L., Chen S., Windham J.P., Chopp M. (1999). Three dimensional measurement of cerebral microvascular plasma perfusion, glial fibrillary acid protein and microtubule associated P-2 immunoreactivity after embolic stroke in rats: A double fluorescent labeled laser scanning confocal microscopic study. Brain Res..

[36] Jung S., Zarow C., Mack W.J., Zheng L., Vinters H.V., Ellis W.G., Lyness S.A., Chui H.C. (2012). Preservation of neurons of the nucleus basalis in subcortical ischemic vascular disease. Arch. Neurol..

[37] Swartz R.H., Sahlas D.J., Black S.E. (2003). Strategic involvement of cholinergic pathways and executive dysfunction: Does location of white matter signal hyperintensities matter?. J. Stroke Cerebrovasc. Dis..

[38] Iadecola C., Yang G., Ebner T.J., Chen G. (1997). Local and propagated vascular responses evoked by focal synaptic activity in cerebellar cortex. J. Neurophysiol..

[39] Salloway S. (2003). Subcortical Vascular Dementia: Binswanger’s and CADASIL.

[40] Pantoni L., Garcia J.H., Gutierrez J.A. (1996). Cerebral white matter is highly vulnerable to ischemia. Stroke.

[41] Schmidt R., Schmidt H., Haybaeck J., Loitfelder M., Weis S., Cavalieri M., Seiler S., Enzinger C., Ropele S., Erkinjuntti T. (2011). Heterogeneity in age-related white matter changes. Acta Neuropathol..

[42] Hommet C., Mondon K., Constans T., Beaufils E., Desmidt T., Camus V., Cottier J.P. (2011). Review of cerebral microangiopathy and Alzheimer’s disease: Relation between white matter hyperintensities and microbleeds. Dement. Geriatr. Cogn. Disord..

[43] Bohnen N.I., Muller M.L.T.M., Kuwabara H., Ocnstantien G.M., Studentski S.A. (2009). Age-associated leukoaraiosis and cortical cholinergic deafferentation. Neurology.

[44] Román G.C. (2004). Brain hypoperfusion: A critical factor in vascular dementia. Neurol. Res..

[45] Zhan S.S., Beyreuther K., Schmitt H.P. (1994). Synaptophysin immunoreactivity of the cortical neuropil in vascular dementia of Binswanger type compared with the dementia of Alzheimer type and non-demented controls. Dementia.

[46] Ahtiluoto S., Polvikoski T., Peltonen M., Solomon A., Tuomilehto J., Winblad B., Sulkava R., Kivipelto M. (2010). Diabetes, Alzheimer disease, and vascular dementia: A population-based neuropathologic study. Neurology.

[47] Borovikova L.V., Ivanova S., Zhang M., Yang H., Botchkina G.I., Watkins L.R., Wang H., Abumrad N., Eaton J.W., Tracey K.J. (2000). Vagus nerve stimulation attenuates the systemic inflammatory response to endotoxin. Nature.

[48] Wang H., Yu M., Ochani M., Amella C.A., Tanovic M., Susarla S., Li J.H., Wang H., Yang H., Ulloa L. (2003). Nicotinic acetylcholine receptor alpha7 subunit is an essential regulator of inflammation. Nature.

[49] Conejero-Goldberg C., Davies P., Ulloa L. (2008). Alpha7 nicotinic acetylcholine receptor: A link between inflammation and neurodegeneration. Neurosci. Biobehav. Rev..

[50] Pavlov V.A., Tracey K.J. (2006). Controlling inflammation: The cholinergic anti-inflammatory pathway. Biochem. Soc. Trans..

[51] Kalaria R.N., Maestre G.E., Arizaga R., Friedland R.P., Galasko D., Hall K., Luchsinger J.A., Ogunniyi A., Perry E.K., Potocnik F. (2008). World Federation of Neurology Dementia Research Group. Alzheimer’s disease and vascular dementia in developing countries: Prevalence, management, and risk factors. Lancet Neurol..

[52] Kim H.J., Moon W.J., Han S.H. (2013). Differential cholinergic pathway involvement in Alzheimer’s disease and subcortical ischemic vascular dementia. J. Alzheimers Dis..

[53] Kim S.H., Kang H.S., Kim H.J., Moon Y., Ryu H.J., Kim M.Y., Han S.H. (2012). The effect of ischemic cholinergic damage on cognition in patients with subcortical vascular cognitive impairment. J. Geriatr. Psychiatry Neurol..

[54] Liu Q., Zhu Z., Teipel S.J., Yang J., Xing Y., Tang Y., Jia J. (2017). White Matter Damage in the Cholinergic System Contributes to Cognitive Impairment in Subcortical Vascular Cognitive Impairment, No Dementia. Front. Aging Neurosci..

[55] Roman G.C., Kalaria R.N. (2006). Vascular determinants of cholinergic deficits in AD and vascular dementia. Neurobiol. Aging.

[56] Low A., Mak E., Rowe J.B., Markus H.S., O’Brien J.T. (2019). Inflammation and cerebral small vessel disease: A systematic review. Ageing Res. Rev..

[57] Tomimoto H., Akiguchi I., Wakita H., Svenaga T., Nakamura S., Kimura J. (1997). Regressive changes of astroglia in white matter lesions in cerebrovascular disease and AD patients. Acta Neuropathol..

[58] O’Brien J.T., Thomas A. (2015). Vascular dementia. Lancet.

[59] Filous A.S., Silver J. (2016). Targeting astrocytes in CNS injury and disease: A translational research approach. Prog. Neurobiol..

[60] Chen A., Akinyemi R.O., Hase Y., Firbank M.J., Ndung’u M.N., Foster V., Craggs L.J., Washida K., Okamoto Y., Thomas A.J. (2016). Frontal white matter hyperintensities, clasmatodendrosis and gliovascular abnormalities in ageing and post-stroke dementia. Brain.

[61] Tong X.K., Hamel E. (1999). Regional cholinergic denervation of cortical microvessels and nitric oxid synthase-containing neurons in AD. Neuroscience.

[62] Cauli B., Tong X.K., Rancillac A., Serluca N., Lambolez B., Rossier J., Hamel E. (2004). Cortical GABA interneurons in neurovascular coupling: Relays for the subcortical vasoactive pathways. J. Neurosci..

[63] Wardlaw J.M., Smith C., Dichgans M. (2013). Mechanism of sporadic cerebral small vessel disease: Insight from neuroimaging. Lancet Neurol..

[64] Englund E.A., Person B. (1987). Correlations between histopathologic white matter changes and proton MR relaxation times in dementia. Alzheimer Dis. Assoc. Disord..

[65] Román G.C. (1987). Senile dementia of the Binswanger type: A vascular form of dementia in the elderly. JAMA.

[66] Vinters H.V., Ellis W.G., Zarow C., Zaias B.W., Jagust W.J., Mack W.J., Chui H.C. (2000). Neuropathological substrate of ischemic vascular dementia. J. Neuropathol. Exp. Neurol..

[67] Moody D.M., Brown W.R., Challa V.R., Anderson R.L. (1995). Periventricular venous collagenosis: Association with leukoaraiosis. Radiology.

[68] Craggs L.J., Hagel C., Kuhlenbaeumer G., Borjesson-Hanson A., Andersen O., Viitanen M., Kalimo H., McLean C.A., Slade J.Y., Hall R.A. (2013). Quantitative vascular pathology and phenotyping familial and sporadic cerebral small vessel diseases. Brain Pathol..

[69] Hainsworth A.H., Oommen A.T., Bridges L.R. (2015). Endothelial Cells and Human Cerebral Small Vessel Disease. Brain Pathol..

[70] Frischer J.M., Pipp I., Stavrou I., Trattnig S., Hainfellner J.A., Knosp E. (2008). Cerebral cavernous malformations: Congruency of histopathological features with the current clinical definition. J. Neurol. Neurosurg. Psychiatry.

[71] Giwa M.O., Williams J., Elderfield K., Jiwa N.S., Bridges L.R., Kalaria R.N., Markus H.S., Esiri M.M., Hainsworth A.H. (2012). Neuropathologic evidence of endothelial changes in cerebral small vessel disease. Neurology.

[72] Zlokovic B.V. (2011). Neurovascular pathways to neurodegeneration in Alzheimer’s disease and other disorders. Nat. Rev. Neurosci..

[73] Cai W., Zhang K., Li P., Zhu L., Xu J., Yang B., Hu X., Lu Z., Chen J. (2017). Dysfunction of the neurovascular unit in ischemic stroke and neurodegenerative diseases: An aging effect. Ageing Res. Rev..

[74] Prisby R.D., Ramsey M.W., Behnke B.J., Dominguez J.M., Donato A.J., Allen M.R., Delp M.D. (2007). Aging reduces skeletal blood flow endothelium dependent vasodilation, and NO bioavailability in Rats. J. Bone Miner. Res..

[75] Nicholson W.T., Vaa B., Hesse C., Eisenach J.H., Joyner M.J. (2009). Aging is associated with reduced prostacyclin-mediated dilation in the human forearm. Hypertenison.

[76] Long D.A., Newaz M.A., Prabahakar S.S., Price K.L., Truong L., Feng L., Mu Oyekan A.O., Johnson R.J. (2005). Loss of nitric oxide and endothelial-derived hyperpolarizing factor-mediated responses in ageing. Kidney Int..

[77] Deplanque D., Lavallee P.C., Labreuche J., Gongora-Rivera F., Jaramillo A., Brenner D., Abboud H., Klein I.F., Touboul P.J., Vicaut E. (2013). Cerebral and extracerebral vasoreactivity in symptomatic lacunar stroke patients: A case-control study. Int. J. Stroke.

[78] Gunarathne A., Patel J.V., Kausar S., Gammon B., Hughes E.A., Lip G.Y. (2009). Glycemic status underlies increased arterial stiffness and impaired endothelial function in migrant South Asian stroke survivors compared to European Caucasians: Pathophysiological insights from the West Birmingham Stroke Project. Stroke.

[79] Markus H.S., Lythgoe D.J., Ostegaard L., O’Sullivan M., Williams S.C. (2000). Reduced cerebral blood flow in white matter in ischaemic leukoaraiosis demonstrated using quantitative exogenous contrast based perfusion MRI. J. Neurol. Neurosurg. Psychiatry.

[80] O’Sullivan M., Lythgoe D.J., Pereira A.C., Summers P.E., Jarosz J.M., Williams S.C., Markus H.S. (2002). Patterns of cerebral blood flow reduction in patients with ischemic leukoaraiosis. Neurology.

[81] Markus H.S., Allan C.L., Ebmeier K.P., Pantoni L., Gorelick P.B. (2014). Cerebral hemodynamics in cerebral small vessel disease. Cerebral Small Vessel Disease.

[82] Van der Loo B., Labugger R., Skepper J.N., BAchschmid M., Kilo J., Powell J.M., Palacios-Callendere M., Erusalimsky J.D., Quaschning T., Malinski T. (2000). Enhanced peroxynitrite formation is associated with vascular ageing. J. Exp. Med..

[83] Puca A.A., Carrizzo A., Ferrario A., Villa F., Vecchione C. (2012). Endothelial nitric oxide synthase, vascular integrity and human exceptional longevity. Immun. Ageing.

[84] Flentje A., Kalsi R., Monahan T.S. (2019). Small GTPases and Their Role in Vascular Disease. Int. J. Mol. Sci..

[85] Hartmann S., Ridley A.J., Lutz S. (2015). The Function of Rho-Associated Kinases ROCK1 and ROCK2 in the Pathogenesis of Cardiovascular Disease. Front. Pharmacol..

[86] Pestonjamasp K., Amieva M.R., Strassel C.P., Nauseef W.M., Furthmayr H., Luna E.J. (1995). Moesin, ezrin, and p205 are actin-binding proteins associated with neutrophil plasma membranes. Mol. Biol. Cell..

[87] Van Nieuw Amerongen G.P., Koolwijk P., Versteilen A., van Hinsbergh V.W. (2003). Involvement of RhoA/Rho kinase signaling in VEGF-induced endothelial cell migration and angiogenesis in vitro. Arter. Thromb. Vasc. Biol..

[88] Matsumoto Y., Uwatoku T., Oi K., Abe K., Hattori T., Morishige K., Eto Y., Fukumoto Y., Nakamura K.I., Shibata Y. (2004). Long-term inhibition of Rho-kinase suppresses neointimal formation after stent implantation in porcine coronary arteries: Involvement of multiple mechanisms. Arter. Thromb. Vasc. Biol..

[89] Szulcek R., Beckers C.M., Hodzic J., de Wit J., Chen Z., Grob T., Musters R.J., Minshall R.D., van Hinsbergh V.W., van Nieuw Amerongen G.P. (2013). Localized RhoA GTPase activity regulates dynamics of endothelial monolayer integrity. Cardiovasc. Res..

[90] Van Nieuw Amerongen G.P., Beckers C.M., Achekar I.D., Zeeman S., Musters R.J., van Hinsbergh V.W. (2007). Involvement of Rho kinase in endothelial barrier maintenance. Arter. Thromb. Vasc. Biol..

[91] Wang J., Liu H., Chen B., Li Q., Huang X., Wang L., Guo X., Huang Q. (2012). RhoA/ROCK-dependent moesin phosphorylation regulates AGE-induced endothelial cellular response. Cardiovasc. Diabetol..

[92] Sun H., Breslin J.W., Zhu J., Yuan S.Y., Wu M.H. (2006). Rho and ROCK signaling in VEGF-induced microvascular endothelial hyperpermeability. Microcirculation.

[93] Gradinaru D., Borsa C., Ionescu C., Prada G.I. (2015). Oxidized LDL and NO synthesis-biomarkers of endothelial dysfunction and ageing. Mech. Ageing Dev..

[94] Cicek F.A., Kandilci H.B., Turan B. (2013). Role of ROCK upregulation in endothelial and smooth muscle vascular functions in diabetic rat aorta. Cardiovasc. Diabetol..

[95] Noma K., Oyama N., Liao J.K. (2006). Physiological role of ROCKs in the cardiovascular system. Am. J. Physiol. Cell Physiol..

[96] Hassan A., Gormley K., O’Sullivan M., Knight J., Sham P., Vallance P., Bamford J. (2004). Markus H Endothelial Nitric Oxide Gene Haplotypes and Risk of Cerebral Small-Vessel Disease. Stroke.

[97] Knottnerus I.L., Cate H., Lodder J., Kessels F., van Oostenbrugge R.J. (2009). Endothelial dysfunction in lacunar stroke: A systematic review. Cerebrovasc. Dis..

[98] Esiri M.M., Wilcock G.K., Morris J.H. (1997). Neuropathological ssessment of the lesions of significance in vascular dementia. J. Neurol. Neurosurg. Psychiatry.

[99] Rajendran P., Rengarajan T., Thangavel J., Nishigaki Y., Sakthisekaran D., Sethi G., Nishigaki I. (2013). The vascular endothelium and human diseases. Int. J. Biol. Sci..

[100] Drake C.T., Iadecola C. (2007). The role of the neuronal signaling in controlling cerebral blood flow. Brain Lang..

[101] Iadecola C. (2013). The pathobiology of vascular dementia. Neuron.

[102] Gallin J.I., Snyderman R. (1999). Inflammation: Basic Principles and Clinical Correlates.

[103] de Leeuw F.E., de Kleine M., Frijns C.J., Fijnheer R., van Gijn J., Kappelle L.J. (2002). Endothelial cell activation is associated with cerebral white matter lesions in patients with cerebrovascular disease. Ann. N. Y. Acad. Sci..

[104] Rouhl R.P., van Oostenbrugge R.J., Theunissen R.O., Knottnerus I.L., Staals J., Henskens L.H. (2010). Autoantibodies against oxidized low-density lipoprotein in cerebral small vessel disease. Stroke.

[105] Wada M., Takahashi Y., Iseki C., Kawanami T., Daimon M., Kato T. (2011). Plasma fibrinogen, global cognitive function, and cerebral small vessel disease: Results of a cross-sectional study in community-dwelling Japanese elderly. Intern. Med..

[106] Knottnerus I.L., Govers-Riemslag J.W., Hamulyak K., Rouhl R.P., Staals J., Spronk H.M. (2010). Endothelial activation in lacunar stroke subtypes. Stroke.

[107] Stevenson S.F., Doubal F.N., Shuler K., Wardlaw J.M. (2010). A systematic review of dynamic cerebral and peripheral endothelial function in lacunar stroke versus controls. Stroke.

[108] Markus H.S., Hunt B., Palmer K., Enzinger C., Schmidt H., Schmidt R. (2005). Markers of endothelial and hemostatic activation and progression of cerebral white matter hyperintensities: Longitudinal results of the Austrian Stroke Prevention Study. Stroke.

[109] Fernando M.S., Simpson J.E., Matthews F., Brayne C., Lewis C.E., Barber R., Kalaria R.N., Forster G., Esteves F., Wharton S.B. (2006). White matter lesions in an unselected cohort of the elderly: Molecular pathology suggests origin from chronic hypoperfusion injury. Stroke.

[110] Tallini Y.N., Brekke J.F., Shui B., Doran R., Hwang S.M., NAkai J., Salama G., Segal S.S., Kotlikoff M.I. (2007). Propagated endothelial Ca++ waves and arteriolar dilatation in vivo: Measurements in Cx40 BAC GCaMP2 transgenic mice. Circ. Res..

[111] Segal S.S. (2015). Integration and modulation of intracellular signaling underlying blood flow control. J. Vasc. Res..

[112] Hen B.P., Kozberg M.G., Bouchard M.B., Shaik M.A., Hillman E.M.C. (2014). A critical role for the vascular endothelium in functional neurovascular coupling in the brain. J. Am. Heart Assoc..

[113] Longden T.A., Hill-Eubanks D.C., Nelosn M.T. (2016). Ion channel networks in the control of cerebral blood flow. J. Cer. Blood Flow. Metab..

[114] Bagher P., Segal S.S. (2011). Regulation of blood flow in the microcirculation: Role of the conducted vasodilation. Acta Physiol..

[115] Uhurovoa H., Kilic K., Tian P., Thunemann M., Desjardins M., Saisan P.A., Sakadžić S., Ness T.V., Mateo C., Cheng Q. (2016). Cell-type specificity of neurovascular coupling in cerebral cortex. ELife.

[116] Longden T.A., Dabertrand F., Koide M., Gonzales A.L., Tykochi N.T., Brayden J.E., Hill-Eubanks D., Nelosn M.T. (2017). Capillary K+ sensing initiates retrograde hyperpolarization to increase local cerebral blood flow. Nat. Neurosci..

[117] Van der Veen P.H., Muller M., Vinken K.L., Hendrikse J., Mali W.P., van der Graaf Y., Geerlings M.I., SMART Study Group (2015). Longitudinal relationship between cerebral small vessel disease and cerebral blood flow. The second manifestations of arterial disease-magnetic resonance study. Stroke.

[118] Gouw A.A., van Der Flier W.M., Fazekas F., van Straaten E.C., Pantoni L., Poggesi A., Inzitari D., Erkinjuntti T., Wahlund L.O., Waldemar G. (2008). Progression of white matter hyperintensities and incidence of new lacunes over a 3-year period: The leukoaraiosis and disability study. Stroke.

[119] Schmidt R., Seiler S., Loitfelder M. (2016). Longitudinal change of small vessel disease related brain abnormalities. J. Cerebr. Blood Flow Metab..

[120] Munoz-Maniega S., Chappell F.M., Valdes-Henrandez M.C., Armitage P.A., Makin S.D., Heye A.K., Thrippleton M.J., Sakka E., Shuler K., Dennis M.S. (2016). Integrity of normal appearing white matter: Influence of age, visible lesion burden and hypertension in patients with small-vessel disease. J. Cerebr. Blood Flow Metab..

[121] Smallwood A., Oulhaj A., Joachim C., Christie S., Sloan C., Smith A.D., Esiri M. (2012). Cerebral subcortical small vessel disease and its relation to cognition in elderly subjects: A pathological study in the Oxford Project to Investigate Memory and Ageing (OPTIMA) cohort. Neuropathol. Appl. Neurobiol..

[122] Kramer J.H., Reed B.R., Mungas D., Weiner M.W., Chui H. (2002). Executive dysfunction in subcortical ischaemic vascular disease. J. Neurol. Neurosurg. Psychiatr..

[123] Burton E., Ballard C., Stephens S., Kenny R.A., Kalaria R., Barber R., O’Brien J. (2003). Hyperintensities and fronto-subcortical atrophy on MRI are substrates of mild cognitive deficits after stroke. Dement. Geriatr. Cogn. Disord..

[124] Cheng B., Golsari A., Fiehler J., Rosenkranz M., Gerloff C., Thomalla G. (2010). Dynamics of regional distribution of ischemic lesions in middle cerebral artery trunk occlusion relates to collateral circulation. J. Cereb. Blood Flow Metab..

[125] Dijkhuizen R.M., Knollema S., van der Worp H.B., Ter Horst G.J., De Wildt D.J., Berkelbach van der Sprenkel J.W., Tulleken K.A., Nicolay K. (1998). Dynamics of cerebral tissue injury and perfusion after temporary hypoxia-ischemia in the rat: Evidence for region-specific sensitivity and delayed damage. Stroke.

[126] Garcia J.H., Liu K.F., Ye Z.R., Gutierrez J.A. (1997). Incomplete infarct and delayed neuronal death after transient middle cerebral artery occlusion in rats. Stroke.

[127] Konaka K., Miyashita K., Naritomi H. (2007). Changes in diffusion-weighted magnetic resonance imaging findings in the acute and subacute phases of anoxic encephalopathy. J. Stroke Cerebrovasc. Dis..

[128] Ravens J.R. (1978). Vascular changes in the human senile brain. Adv. Neurol..

[129] Klassen A.C., Sung J.H., Stadlan E.M. (1968). Histological changes in cerebral arteries with increasing age. J. Neuropathol. Exp. Neurol..

[130] Cummings J.L. (1993). Frontal-subcortical circuits and human behavior. Arch. Neurol..

[131] Mega M.S., Cummings J.L. (1994). Frontal-subcortical circuits and neuropsychiatric disorders. J. Neuropsychiatry Clin. Neurosci..

[132] Smith A.D., Refsum H. (2016). Homocysteine, B vitamins, and cognitive impairment. Annu. Rev. Nutr..

[133] Blom H.J., Smulders Y. (2011). Overview of homocysteine and folate metabolism. With special references to cardiovascular disease and neural tube defects. J. Inherit. Metab. Dis..

[134] Loscalzo J., Handy D.E. (2014). Epigenetic modifications: Basic mechanisms and role in cardiovascular disease. 2013 Grover Conference Series. Pulm. Circ..

[135] Miles L., Allen E., Mills K., Clarke R., Uauy R., Dangour A.D. (2016). Vitamin B12 status and neurologic function in older people: A cross-sectional analysis of baseline trial data from the Older People and Enhanced Neurological Function (OPEN) study. Am. J. Clin. Nutr..

[136] Obeid R., Herrmann W. (2006). Mechanisms of homocysteine neurotoxicity in neurodegenerative diseases with special reference to dementia. FEBS Lett..

[137] Price B.R., Wilcock D.M., Weekman E.M. (2018). Hyeprhomocysteinemia as a risk factor for vascular contributions to cognitive impairment and dementia. Front. Aging Neurosci..

[138] Mudd S.H., Cantoni G.L. (1958). Activation of methionine for transmethylation. III. The methionine-activating enzyme of Bakers’ yeast. J. Biol. Chem..

[139] Mato J.M., Alvarez L., Ortiz P., Pajares M.A. (1997). S-adenosylmethionine synthesis: Molecular mechanisms and clinical implications. Pharmacol. Ther..

[140] Taha S., Azzi A., Ozer N.K. (1999). Homocysteine induces DNA synthesis and proliferation of vascular smooth muscle cells by a hydrogen peroxide-independent mechanism. Antioxid. Redox Signal..

[141] Robinson J.L., McBreairty L.E., Randell E.W., Harding S.V., Bartlett R.K., Brunton J.A., Bertolo R.F. (2018). Betaine or folate can equally furnish remethylation to methionine and increase transmethylation in methionine-restricted neonates. J. Nutr. Biochem..

[142] Kotb M., Mudd S.H., Mato J.M. (1997). Consensus nomenclature for the mammalian methionine adenosyltransferase genes and gene products. Trends Genet..

[143] Smulders Y.M., Blom H.J. (2011). The homocysteine controversy. J. Inherit. Metab. Dis..

[144] Selhub J. (1999). Homocysteine metabolism. Annu. Rev. Nutr..

[145] Parnetti L., Bottiglieri T., Lowenthal D. (1997). Role of homocysteine in age-related vascular and non-vascular diseases. Aging Clin. Exp. Res..

[146] Handy D.E., Castro R., Loscalzo J. (2011). Epigenetic modifications: Basic mechanisms and role in cardiovascular disease. Circulation.

[147] Enk C., Hougaard K., Hippe E. (1980). Reversible dementia and neuropathy associated with folate deficiency 16 years after partial gastrectomy. Scand. J. Haematol..

[148] Bottiglieri T. (1997). Ademetionine (S-adenosylmethionine) neuropharmacology: Implications for drug therapies in psychiatric and neurological disorders. Expert Opin. Investig. Drugs.

[149] Weir D.G., Keating S., Molloy A. (1988). Methylation deficiency causes vitamin B12-associated neuropathy in the pig. J. Neurochem..

[150] Surtees R., Leonard J., Austin S. (1991). Association of demyelination with deficiency of cerebrospinal-fluid S-adenosylmethionine in inborn errors of methyl-transfer pathway. Lancet.

[151] Pennypacker L.C., Allen R.H., Kelly J.P. (1992). High prevalence of cobalamin deficiency in elderly outpatients. J. Am. Geriatr. Soc..

[152] McKeever M.P., Weir D.G., Molloy A., Scott J.M. (1991). Betaine-homocysteine methyltransferase: Organ distribution in man, pig and rat and subcellular distribution in the rat. Clin. Sci..

[153] Leclerc D., Wilson A., Dumas R. (1998). Cloning and mapping of a cDNA for methionine synthase reductase, a flavoprotein defective in patients with homocystinuria. Proc. Natl. Acad. Sci. USA.

[154] Sunden S.L., Renduchintala M.S., Park E.I., Miklasz S.D., Garrow T.A. (1997). Betaine-homocysteine methyltransferase expression in porcine and human tissues and chromosomal localization of the human gene. Arch. Biochem. Biophys..

[155] Quéré I., Paul V., Rouillac C. (1999). Spatial and temporal expression of the cystathionine beta-synthase gene during early human development. Biochem. Biophys. Res. Commun..

[156] Pietrzik K., Bronstrup A. (1998). Vitamins B12, B6 and folate as determinants of homocysteine concentration in the healthy population. Eur. J. Pediatr..

[157] Huang Y.C., Chang S.J., Chiu Y.T., Chang H.H., Cheng C.H. (2003). The status of plasma homocysteine and related B-vitamins in healthy young vegetarians and nonvegetarians. Eur. J. Nutr..

[158] Kulkarni K., Richard B.C. (2003). Lifestyle, homocysteine and the metabolic syndrome. Metab. Syndr. Relat. Disord..

[159] Ansari R., Mahta A., Mallack E., Luo J.J. (2014). Hyperhomocysteinemia and neurologic disorders: A review. J. Clin. Neurol..

[160] Stea T.H., MAnsoor M.A., Wandel M., Uglem S., Frolich W. (2008). Changes in predictors and status of homocysteine in young male adults after dietary intervention with vegetables, fruits and bread. Eur. J. Nutr..

[161] Pushpakumar S., Kundu S., Sen U. (2014). Endothelial dysfunction: The link between homocysteine and hydrogen sulfide. Curr. Med. Chem..

[162] Moretti R., Dal Ben M., Gazzin S., Tiribelli C. (2017). Homcysteine in neurology: From endothelium to neurodegeneration. Curr. Nutr. Food Sci..

[163] Surtees R., Bowron A., Leonard J. (1997). Cerebrospinal fluid and plasma total homocysteine and related metabolites in children with cystathionine beta-synthase deficiency: The effect of treatment. Pediatr. Res..

[164] Afman L.A., Blom H.J., Drittij M.J., Brouns M.R., van Straaten H.W. (2005). Inhibition of transmethylation disturbs neurulation in chick embryos. Brain Res. Dev. Brain Res..

[165] Kamath A.F., Chauhan A.K., Kisucka J. (2006). Elevated levels of homocysteine compromise blood-brain barrier integrity in mice. Blood.

[166] Troen A.M. (2005). The central nervous system in animal models of hyperhomocysteinemia. Prog. NeuroPsychopharmacol. Biol. Psychiatry.

[167] Algaidi S.A., Christie L.A., Jenkinson A.M. (2006). Long-term homocysteine exposure induces alterations in spatial learning, hippocampal signalling and synaptic plasticity. Exp. Neurol..

[168] Ganguly P., Alam S.F. (2015). Role of homocysteine in the development of cardiovascular disease. Nutr. J..

[169] Sultan M.O., Farooque U., Javed R., Khan M.I., Karimi S., Abdul Sattar R., Cheema O. (2020). Correlation of Homocysteine Level and Age in Patients with Ischemic Stroke. Cureus.

[170] Moretti R., Peinkhofer C. (2019). B Vitamins and Fatty Acids: What Do They Share with Small Vessel Disease-Related Dementia?. Int. J. Mol. Sci..

[171] Moretti R. (2019). Homocysteine: New Aspects of an Ancient Enigma. Cardiology.

[172] Piao X., Wu G., Yang P., Shen J., De A., Wu J., Qu Q. (2018). Association between Homocysteine and Cerebral Small Vessel Disease: A Meta-Analysis. J. Stroke Cerebrovasc. Dis..

[173] Rutten-Jacobs L.C.A., Traylor M., Adib-Samii P., Thijs V., Sudlow C., Rothwell P.M., Boncoraglio G., Dichgans M., Meschia J., Maguire J. (2016). Association of MTHFR C677T Genotype With Ischemic Stroke Is Confined to Cerebral Small Vessel Disease Subtype. Stroke.

[174] Irizarry M.C., Gurol M.E., Raju S. (2005). Association of homocysteine with plasma amyloid beta protein in aging and neurodegenerative disease. Neurology.

[175] Hasegawa T., Ukai W., Jo D.-G. (2005). Homocysteic acid induces intraneuronal accumulation of neurotoxic Abeta42, implications for the pathogenesis of Alzheimer’s disease. J. Neurosci. Res..

[176] Morris M.S. (2003). Homocysteine and Alzheimer’s disease. Lancet Neurol..

[177] Kruman I.I., Kumaravel T.S., Lohani A. (2002). Folic acid deficiency and homocysteine impair DNA repair in hippocampal neurons and sensitize them to amyloid toxicity in experimental models of Alzheimer’s disease. J. Neurosci..

[178] Sai X., Kawamura Y., Kokame K. (2002). Endoplasmic reticulum stress-inducible protein, Herp, enhances presenilin-mediated generation of amyloid beta-protein. J. Biol. Chem..

[179] Selkoe D.J. (2001). Presenilin, Notch, and the genesis and treatment of Alzheimer’s disease. Proc. Natl. Acad. Sci. USA.

[180] Scarpa S., Fuso A., D’Anselmi F., Cavallaro R.A. (2003). Presenilin 1 gene silencing by S-adenosylmethionine: A treatment for Alzheimer disease?. FEBS Lett..

[181] Baernstein H.D. (1934). A modification of the method for determining methionine in proteins. J. Biol. Chem..

[182] Jakubowski H., Fersht A. (1981). Alternative pathways of rejection of noncognate amino acids by aminoacyl-tRNA synthetases. Nucleic Acids Res..

[183] Jakubowski H. (1990). Proofreading in vivo: Editing of homocysteine by methionyl-tRNA synthetase in Escherichia *coli*. Proc. Natl. Acad. Sci. USA.

[184] Jakubowski H. (1997). Metabolism of homocysteine thiolactone in human cell cultures: Possible mechanism for pathological consequences of elevated homocysteine levels. J. Biol. Chem..

[185] Sharma G.S., Kumar T., Dar T.A., Singh L.R. (2015). Protein-N-Homocysteinylation: Form cellular toxicity to neurodegeneration. Biochim. Et Biophys. Acta.

[186] Jakubowski H. (2000). Homocysteine Thiolactone: Metabolic Origin and Protein Homocysteinylation in Humans. J. Nutr..

[187] Jakubowski H. (2002). Homocysteine is a protein amino acid in humans. Implications for homocysteine-linked disease. J. Biol. Chem..

[188] Jakubowski H. (1999). Protein homocysteinylation: Possible mechanism underlying pathological consequences of elevated homocysteine levels. FASEB J..

[189] Sikora M., Marczak Ł., Kubalska J., Graban A., Jakubowski H. (2014). Identification of N-homocysteinylation sites in plasma proteins. Amino Acids.

[190] Jacovina A.T., Deora A.B., Ling Q., Broekman M.J., Almeida D., Greenberg C.B., Marcus A.J., Smith J.D., Hajjar K.A. (2009). Homocysteine inhibits neoangiogenesis in mice through blockade of annexin A2-dependent fibrinolysis. J. Clin. Investig..

[191] Lim A., Sengupta S., McComb M.E., Théberge R., Wilson W.G., Costello C.E., Jacobsen D.W. (2003). In vitro and in vivo interactions of homocysteine with human plasma transthyretin. J. Biol. Chem..

[192] Jakubowski H. (2019). Homocysteine Modification in Protein Structure/Function and Human Disease. Physiol. Rev..

[193] Hortin G.L., Seam N., Hoehn G.T. (2006). Bound homocysteine, cysteine, and cysteinylglycine distribution between albumin and globulins. Clin. Chem..

[194] Jakubowski H. (2013). Homocysteine in Protein Structure/Function and Human Disease—Chemical Biology of Homocysteine-Containing Proteins.

[195] Lai W.K., Kan M.Y. (2015). Homocysteine-Induced Endothelial Dysfunction. Ann. Nutr. Metab..

[196] Perla-Kajan J., Twardowski T., Jakubowski H. (2007). Mechanisms of homocysteine toxicity in humans. Amino Acids.

[197] Frey D., Braun O., Briand C., Vasak M., Grutter M.G. (2006). Structure of the mammalian NOS regulator dimethylarginine dimethylaminohydrolase: A basis for the design of specific inhibitors. Structure.

[198] Leulliot N., Quevillon-Cheruel S., Sorel I. (2004). Structure of protein phosphatase methyltransferase 1 (PPM1), a leucine carboxyl methyltransferase involved in the regulation of protein phosphatase 2A activity. J. Biol. Chem..

[199] Ferreira A., Lu Q., Orecchio L., Kosik K.S. (1997). Selective phosphorylation of adult tau isoforms in mature hippocampal neurons exposed to fibrillar A beta. Mol. Cell Neurosci..

[200] Wang J.Z., Gong C.X., Zaidi T., Grundke-Iqbal I., Iqbal K. (1995). Dephosphorylation of Alzheimer paired helical filaments by protein phosphatase-2A and -2B. J. Biol. Chem..

[201] Vogelsberg-Ragaglia V., Schuck T., Trojanowski J.Q., Lee V.M. (2001). PP2A mRNA expression is quantitatively decreased in Alzheimer’s disease hippocampus. Exp. Neurol..

[202] Sontag E., Hladik C., Montgomery L. (2004). Downregulation of protein phosphatase 2A carboxyl methylation and methyltransferase may contribute to Alzheimer disease pathogenesis. J. Neuropathol. Exp. Neurol..

[203] Zhao W.-Q., Feng C., Alkon D.L. (2003). Impairment of phosphatase 2A contributes to the prolonged MAP kinase phosphorylation in Alzheimer’s disease fibroblasts. Neurobiol. Dis..

[204] Vafai S.B., Stock J.B. (2002). Protein phosphatase 2A methylation: A link between elevated plasma homocysteine and Alzheimer’s Disease. FEBS Lett..

[205] Tolstykh T., Lee J., Vafai S., Stock J.B. (2000). Carboxyl methylation regulates phosphoprotein phosphatase 2A by controlling the association of regulatory B subunits. EMBO J..

[206] Ho P.I., Ashline D., Dhitavat S. (2003). Folate deprivation induces neurodegeneration: Roles of oxidative stress and increased homocysteine. Neurobiol. Dis..

[207] Wuerthele S.E., Yasuda R.P., Freed W.J., Hoffer B.J. (1982). The effect of local application of homocysteine on neuronal activity in the central nervous system of the rat. Life Sci..

[208] Lipton S.A., Kim W.K., Choi Y.B. (1997). Neurotoxicity associated with dual actions of homocysteine at the N-methyl-D-aspartate receptor. Proc. Natl. Acad. Sci. USA.

[209] Ito S., Provini L., Cherubini E. (1991). L-homocysteic acid mediates synaptic excitation at NMDA receptors in the hippocampus. Neurosci. Lett..

[210] Klancnik J.M., Cuénod M., Gähwiler B.H., Jiang Z.P., Do K.Q. (1992). Release of endogenous amino acids, including homocysteic acid and cysteine sulphinic acid, from rat hippocampal slices evoked by electrical stimulation of Schaffer collateral-commissural fibres. Neuroscience.

[211] Kim J.P., Koh J.Y., Choi D.W. (1987). L-homocysteate is a potent neurotoxin on cultured cortical neurons. Brain Res..

[212] Ziemiffska E., Stafiej A., Lazarewicz J.W. (2003). Role of group I metabotropic glutamate receptors and NMDA receptors in homocysteine-evoked acute neurodegeneration of cultured cerebellar granule neurones. Neurochem. Int..

[213] Shi Q., Savage J.E., Hufeisen S.J. (2003). L-homocysteine sulfinic acid and other acidic homocysteine derivatives are potent and selective metabotropic glutamate receptor agonists. J. Pharmacol. Exp..

[214] Robert K., Pagès C., Ledru A. (2005). Regulation of extracellular signal-regulated kinase by homocysteine in hippocampus. Neuroscience.

[215] De Lau L.M., Koudstaal P.J., van Meurs J.B., Uitterlinden A.G., Hofman A., Breteler M.M. (2005). Methylenterahydrofolate reductase C677T genotype and PD. Annu. Neurol..

[216] Zhao P., Yang J.F., Liu W., Wang Y., Sun Y.N., Li Q. (2013). Effects of entacapone on plasma homocysteine in Parkinson’s Disease patients on levodopoa. Zhongha Yi Xue Za Zhi.

[217] Mok S.S., Turner B.J., Beyreuther K. (2002). Toxicity of substrate-bound amyloid peptides on vascular smooth muscle cells is enhanced by homocysteine. Eur. J. Biochem. FEBS.

[218] Pang X., Liu J., Zhao J., Mao J., Zhang X., Feng L. (2014). Homocysteine induces the expression of C-reactive protein via NMDAr-ROS-MAPK-NF-KB signal pathway in rat vascular smooth muscle cells. Atherosclerosis.

[219] Nelson A.R., Sweeney M.D., Sagare A.P., Zlokovic B.V. (2016). Neurovascular dysfunction and neurodegeneration in dementia and Alzheimer’s disease. Biochim. Biophys. Acta.

[220] Jakubowski H. (2008). The pathophysiological hypothesis of homocysteine thiolactone-mediated vascular disease. J. Physiol. Pharmacol..

[221] Mercie P., Garnier O., Lascoste L., Renard M., Closse C., Durrieu F., Marit G., Boisseau R.M., Belloc F. (2000). Homocysteine-thiolactone induces caspase-independent vascular endothelial cell death with apoptotic features. Apoptosis.

[222] Dayal S., Wilson K.M., Leo L., Arning E., Bottiglieri T., Lentz S.R. (2006). Enhanced susceptibility to arterial thrombosis in a murine model of hyperhomocysteinemia. Blood.

[223] Undas A., Brozek J., Szczeklik A. (2005). Homocysteine and thrombosis: From basic science to clinical evidence. Thromb. Haemost..

[224] Sauls D.L., Lockhart E., Warren M.E., Lenkowski A., Wilhelm S.E., Hoffman M. (2006). Modification of fibrinogen by homocysteine thiolactone increases resistance to fibrinolysis: A potential mechanism of the thrombotic tendency in hyperhomocysteinemia. Biochemistry.

[225] Tamura Y., Inoue A., Ijiri Y., Naemura A., Yamamoto J. (2014). Short- and long-term treatment with folic acid suppresses thrombus formation in atherogenic mice in vivo. Pathophysiology.

[226] Perna A.F., Ingrosso D., De Santo N.G. (2003). Homocysteine and oxidative stress. Amino Acids.

[227] Petras M., Tatarakova Z., Kovalska M., Mokra D., Dobrota D., Lehotsky J., Drgova A. (2014). Hyperhomocysteinemia as a risk factor for the neuronal system disorders. J. Physiol. Pharmacol..

[228] Wyse A.T.S., Zugno A.I., Streck E.L. (2002). Inhibition of Na(+), K(+)-ATPase activity in hippocampus of rats subjected to acute administration of homocysteine is prevented by vitamins E and C treatment. Neurochem. Res..

[229] Bleie O., Semb A.G., Grundt H. (2007). Homcysteine-lowering therapy does not affect inflammatory markers of atherosclerosis in patients with stable coronary disease. J. Int. Med..

[230] Ploder M., Kurz K., Splitter A., Neurauter G., Roth E., Fuch D. (2010). Early increase of plasma Hcy in sepsis patients with poor outcome. Mol. Med..

[231] Li J.-J., Li Q., Du H.-P. (2015). Homocysteine Triggers inflammatory responses in macrophages through inhibiting CSE-H2S signaling via DNA hypermethylation of CSE promoter. Int. J. Mol. Sci..

[232] Krishna S.M., Dear A., Craig J.M., Norman P.E., Golledge J. (2013). The potential role of homocysteine mediated DNA methylation and associated epigenetic changes in abdominal aortic aneurysm formation. Atherosclerosis.

[233] Yi-Deng J., Tao S., Hui-Ping Z. (2007). Folate and ApoE DNA methylation induced by homocysteine in human monocytes. DNA Cell Biol..

[234] Chang P.-Y., Lu S.-C., Lee C.-M. (2008). Homocysteine inhibits arterial endothelial cell growth through transcriptional downregulation of fibroblast growth factor-2 involving G protein and DNA methylation. Circ. Res..

[235] Kamat P.K., Kalani A., Givvimani S., Sathnur P.B., Tyagi S.C., Tyagi N. (2013). Hydrogen sulfide attenuates neurodegeneration and neurovascular dysfunction induced by intracerebral-administered homocysteine in mice. Neuroscience.

[236] Han S., Wu H., Li W., Gao P. (2015). Protective effects of genistein in homocysteine-induced endothelial cell inflammatory injury. Mol. Cell Biochem..

[237] Li J., Luo M., Xie N., Wang J., Chen L. (2016). Curcumin protects endothelial cells against homocysteine induced injury through inhibiting inflammation. Am. J. Transl. Res..

[238] Keegan P.M., Wilder C.L., Platt M.O. (2012). Tumor necrosis factor alpha stimulates cathepsin K and V activity via juxtacrine monocyteendothelial cell signaling and JNK activation. Mol. Cell Biochem..

[239] Du X., Chen N.L., Wong A., Craik C.S., Brömme D. (2013). Elastin degradation by cathepsin V requires two exosites. J. Biol. Chem..

[240] Li X., Cheng X.W., Hu L., Wu H., Guo-Ping Hao C.N., Jiang H., Zhu E., Huang Z., Inoue A., Sasaki T. (2015). Cathepsin S activity controls ischemia-induced neovascularization in mice. Int. J. Cardiol..

[241] Zhou J., Zhang Y.Y., Li Q.Y., Cai Z.H. (2015). Evolutionary history of cathepsin L (L-like) family genes in vertebrates. Int. J. Biol. Sci..

[242] Pribis J.P., Al-Abed Y., Yang H., Gero D., Xu H., Montenegro M.F., Bauer E.M., Kim S., Chavan S.S., Cai C. (2015). The HIV protease inhibitor saquinavir inhibits HMGB1 driven inflammation by targeting the interaction of cathepsin V with TLR4/MyD88. Mol. Med..

[243] Reichenbach G., Starzinski-Powitz A., Sloane B.F., Doll M., Kippenberger S., Bernd A., Kaufmann R., Meissner M. (2013). PPARα agonist Wy14643 suppresses cathepsin B in human endothelial cells via transcriptional, post-transcriptional and post-translational mechanisms. Angiogenesis.

[244] Platt M.O., Shockey W.A. (2016). Endothelial cells and cathepsins: Biochemical and biomechanical regulation. Biochimie.

[245] Leng Y.P., Ma Y.S., Li X.G., Chen R.F., Zeng P.Y., Li X.H., Qiu C.F., Li Y.P., Zhang Z., Chen A.F. (2018). l-Homocysteine-induced cathepsin V mediates the vascular endothelial inflammation in hyperhomocysteinaemia. Br. J. Pharmacol..

[246] Ahmad S., Siddiqi M.I. (2017). Insights from molecular modeling into the selective inhibition of cathepsin S by its inhibitor. J. Mol. Model..

[247] Aavik E., Lumivuori H., Leppänen O., Wirth T., Häkkinen S.K., Bräsen J.H., Beschorner U., Zeller T., Braspenning M., van Criekinge W. (2015). Global DNA methylation analysis of human atherosclerotic plaques reveals extensive genomic hypomethylation and reactivation at imprinted locus 14q32 involving induction of a miRNA cluster. Eur. Heart J..

[248] Boldyrev A., Bryshkova E., MAshkina A., Vladychenskaya E. (2013). Why is homocysteine toxic for the nervous and immune systems?. Curr. Aging Sci..

[249] Essouma M., Noubiap J.J.N. (2015). Therapeutic potential of folic acid supplementation for cardiovascular disease prevention through homocysteine lowering and blockade in rheumatoid arthritis patients. Biomark. Res..

[250] Ying G., Wang Y., Cen X.M., Yang M., Liang Y., Xie Q.B. (2015). Lipid peroxidation-mediated inflammation promotes cell apoptosis through activation of NFK-B pathway in rheumatoid arthritis synovial cells. Med. Infalmm..

[251] Deng J., Lu S., Li H. (2017). Homocysteine activates B cells via regulating PKM-2 dependent metabolic reprogramming. J. Immunol..

[252] Antoniades C., Tousoulis D., Marinou K. (2006). Asymmetrical dimethylarginine regulates endothelial function in methionine-induced but not in chronic homocystinemia in humans: Effect of oxidative stress and proinflammatory cytokines. Am. J. Clin. Nutr..

[253] Schwedhelm E., Xanthakis V., Maas R. (2009). Asymmetric dimethylarginine reference intervals determined with liquid chromatography–tandem mass spectrometry: Results from the Framingham offspring cohort. Clin. Chem..

[254] Li Z., Sun L., Zhang H. (2003). Elevated plasma homocysteine was associated with hemorrhagic and ischemic stroke, but methylenetetrahydrofolate reductase gene c677t polymorphism was a risk factor for thrombotic stroke a multicenter case-control study in China. Stroke.

[255] Kumar A., Palfrey H.A., Pathak R., Kadowitz P.J., Gettys T.W., Murthy S.N. (2017). The metabolism and significance of homocysteine in nutrition and health. Nutr. Metab..

[256] Li J.G., Chu J., Barrero C., Merali S., Praticò D. (2014). Homocysteine exacerbates β-amyloid pathology, tau pathology, and cognitive deficit in a mouse model of Alzheimer disease with plaques and tangles. Ann. Neurol..

[257] Vallance P., Leiper J. (2004). Cardiovascular biology of the asymmetric dimethylarginine: Dimethylarginine dimethylaminohydrolase pathway. Arterioscler. Thromb. Vasc. Biol..

[258] Lentz S.R., Rodionov R.N., Dayal S. (2003). Hyperhomocysteinemia, endothelial dysfunction, and cardiovascular risk: The potential role of ADMA. Atheroscler. Suppl..

[259] Dayal S., Lentz S.R. (2005). ADMA and hyperhomocysteinemia. Vasc. Med..

[260] Li T., Huang Y., Cai W., Chen X., Men X., Lu T., Wu A., Lu Z. (2020). Age-related cerebral small vessel disease and inflammaging. Cell Death Dis..

[261] Hassan A., Hunt B.J., O’Sullivan M., Bell R., D’Souza R., Jeffery S., Bamford J.M., Markus H.S. (2004). Homocysteine is a risk factor for cerebralr small vessel disease, acting via endothelial dysfunction. Brain.

[262] Schroder M., Kaufman R.J. (2005). ER stress and the unfolded protein response. Mutat. Res..

[263] Walter P., Ron D. (2011). The unfolded protein response: From stress pathway to homeostatic regulation. Science.

[264] Tian X., Zhao L., Song X., Yan Y., Liu N., Li T., Yan B., Liu B. (2016). HSP27 inhibits homocysteien-induced endothelial apoptosis by modulation of ROS production and mithocondrial caspase-depndent apoptotic pathway. Biomed Res. Int..

[265] Hossain G.S., van Thienen J.V., Werstuck G.H., Zhou J., Sood S.K., Dickhout J.G., de Koning A.B., Tang D., Wu D., Falk E. (2003). TDAG51 is induced by homocysteine, promotes detachment-mediated programmed cell death, and contributes to the development of atherosclerosis in hyperhomocysteinemia. J. Biol. Chem..

[266] Hofmann M.A., Lalla E., Lu Y., Gleason M.R., Wolf B.M., Tanji N., Ferran LJJr Kohl B., Rao V., Kisiel W., Stern D.M. (2001). Hyperhomocysteinemia enhances vascular inflammation and accelerates atherosclerosis in a murine model. J. Clin. Investig..

[267] McCully K.S. (2009). Chemical pathology of homocysteine. IV. Excitotoxicity, oxidative stress, endothelial dysfunction, and inflammation. Ann. Clin. Lab. Sci..

[268] Li T., Chen Y., Li J., Yang X., Zhang H., Qin X., Hu Y., Mo Z. (2015). Serum Homocysteine Concentration Is Significantly Associated with Inflammatory/Immune Factors. PLoS ONE.

[269] Reddy V.S., Trinath J., Reddy G.B. (2019). Implication of homocysteine in protein quality control processes. Biochimie.

[270] Ai Y., Sun Z., Peng C., Liu L., Xiao X., Li J. (2017). Homocysteine Induces Hepatic Steatosis Involving ER Stress Response in High Methionine Diet-Fed Mice. Nutrients.

[271] Yakub M., Schulze K.J., Khatry S.K., Stewart C.P., Christian P., West K.P. (2014). High plasma homocysteine increases risk of metabolic syndrome in 6 to 8 year old children in rural Nepal. Nutrients.

[272] Zheng X., Xu F., Liang H., Cao H., Cai M., Xu W., Weng J. (2017). SIRT1/HSF1/HSP pathway is essential for exenatide-alleviated, lipid-induced hepatic endoplasmic reticulum stress. Hepatology.

[273] Chen S., Dong Z., Cheng M., Zhao Y., Wang M., Sai N., Wang X., Liu H., Huang G., Zhang X. (2017). Homocysteine exaggerates microglia activation and neuroinflammation through microglia localized STAT3 overactivation following ischemic stroke. J. Neuroinflammation.

[274] Raible D.J., Frey L.C., Brooks-Kayal A.R. (2014). Effects of JAK2-STAT3 signaling after cerebral insults. JAKSTAT.

[275] Liang Z., Wu G., Fan C., Xu J., Jiang S., Yan X., Di S., Ma Z., Hu W., Yang Y. (2016). The emerging role of signal transducer and activator of transcription 3 in cerebral ischemic and hemorrhagic stroke. Prog. Neurobiol..

[276] Zhu H., Zou L., Tian J., Du G., Gao Y. (2013). SMND-309, a novel derivative of salvianolic acid B, protects rat brains ischemia and reperfusion injury by targeting the JAK2/STAT3 pathway. Eur. J. Pharmacol..

[277] Satriotomo I., Bowen K.K., Vemuganti R. (2006). JAK2 and STAT3 activation contributes to neuronal damage following transient focal cerebral ischemia. J. Neurochem..

[278] Yi J.H., Park S.W., Kapadia R., Vemuganti R. (2007). Role of transcription factors in mediating post-ischemic cerebral inflammation and brain damage. Neurochem. Int..

[279] Probert L., Akassoglou K., Pasparakis M., Kontogeorgos G., Kollias G. (1995). Spontaneous inflammatory demyelinating disease in transgenic mice showing central nervous system-specific expression of tumor necrosis factor alpha. Proc. Natl. Acad. Sci. USA.

[280] Wu X., Zhang L., Miao Y., Yang J., Wang X., Wang C.C., Feng J., Wang L. (2019). Homocysteine causes vascular endothelial dysfunction by disrupting endoplasmic reticulum redox homeostasis. Redox Biol..

[281] Ji C., Kaplowitz N. (2004). Hyperhomocysteinemia, endoplasmic reticulum stress, and alcoholic liver injury. World J. Gastroenterol..

[282] Hohsfeld L.A., Humpel C. (2010). Homocysteine enhances transmigration of rat monocytes through a brain capillary endothelial cell monolayer via ICAM-1. Curr. Neurovasc. Res..

[283] Gutteridge J.M., Halliwell B. (2010). Antioxidants: Molecules, medicines, and myths. Biochem. Biophys. Res. Commun..

[284] Thampi P., Stewart B.W., Joseph L., Melnyk S.B., Hennings L.J., Nagarajan S. (2008). Dietary homocysteine promotes atherosclerosis in apoE‑deficient mice by inducing scavenger receptors expression. Atherosclerosis.

[285] Trujillo M.B.A., Souza J.M., Romero N., Castro L., Thomson L., Radi R., Ignaro L. (2010). Mechanisms and Biological Consequences of Peroxynitrite-Dependent Protein Oxidation and Nitration. Nitric Oxide. Biology and Pathobiology, 2nd edition.

[286] Gao H.M., Zhou H., Hong J.S. (2012). NADPH oxidases: Novel therapeutic targets for neurodegenerative diseases. Trends Pharmacol. Sci..

[287] Paravicini T.M., Touyz R.M. (2008). NADPH Oxidases, Reactive Oxygen Species, and Hypertension. Diabetes Care.

[288] Yang Q., He G.W. (2019). Imbalance of Homocysteine and H_2_S: Significance, Mechanisms, and Therapeutic Promise in Vascular Injury. Oxid. Med. Cell Longev..

[289] Ma S.-C., Hao Y.-J., Jiao Y., Wang Y.-H., Xu L.-B., Mao C.-Y., Yang X.-L., Yang A.-N., Tian J., Zhang M.-H. (2017). Homocysteine‑induced oxidative stress through TLR4/NF‑κB/DNMT1‑mediated LOX‑1 DNA methylation in endothelial cells. Mol. Med. Rep..

[290] Jellinger K.A. (2013). Pathology and pathogenesis of vascular cognitive impairment-a critical update. Front. Aging Neurosci..

[291] Ignarro L.J., Buga G.M., Wood K.S., Byrns R.E., Chaudhuri G. (1987). Endothelium-derived relaxing factor produced and released from artery and vein is nitric oxide. Proc. Natl. Acad. Sci. USA.

[292] Tchantchou F., Goodfellow M., Li F., Ramsue L., Miller C., Puche A., Fiskum G. (2020). Hyperhomocysteinemia-Induced Oxidative Stress Exacerbates Cortical Traumatic Brain Injury Outcomes in Rats. Cell. Mol. Neurobiol..

[293] Hoffman M. (2011). Hypothesis: Hyperhomocysteinemia is an indicator of oxidant stress. Med. Hypotheses.

[294] Sen U., Mishra P.K., Tyagi N., Tyagi S.C. (2010). Homocysteine to hydrogen sulfide or hypertension. Cell Biochem. Biophys..

[295] Sawle P., Foresti R., Green C.J., Motterlini R. (2001). Homocysteine attenuates endothelial heme-oxygenase-1 induction by nitric oxide (NO) and hypoxia. FEBS Lett..

[296] Stuhlinger M.C., Tsao P.S., Her J.H., Kimoto M., Balint R.F., Cooke J.P. (2001). Homocysteine impairs the nitric oxide synthase pathway: Role of asymmetric dimethylarginine. Circulation.

[297] Vallance P., Chan N. (2001). Endothelial function and nitric oxide: Clinical relevance. Heart.

[298] Tyagi N., Sedoris K.C., Steed M., Ovechkin A.V., Moshal K.S., Tyagi S.C. (2005). Mechanisms of homocysteine-induced oxidative stress. Am. J. Physiol-Heart Circ. Physiol..

[299] Fornier I., Ploye F., Cottet-Emard J.M., Brun J., Claustrat B. (2002). Folate deficiency alters melatonin secretion in rats. J. Nutr..

[300] Reiter R.J., Tan D.X., Pappolla M.A. (2004). Melatonin relieves the neural oxidative burden tht contributes to dementias. Annu. N. Y. Acad. Sci..

[301] Baydar G., Ozer M., Yasar A., Tuzcu M., Koz S.T. (2005). Melatonin improves learning and memory performances impaired by hyperhomocysteinemia in rats. Brain Res..

[302] Baydar G., Kutlu S., Nazirroglu M., Canpolat S., Sandal S., Ozcan M., Kelestimur H. (2003). Inhibitory effects of melatonin on neural lipid peroxidation induced by intracerebroventricularly administered homocysteine. J. Pinel. Res..

[303] Curro M., Gugliandolo A., Gangemi C., Risitano R., Ientile R., Caccamo D. (2014). Toxic effects of mildy elevated homocysteine concnetrations in neuronal-like cells. Neurochem. Res..

[304] Sharma M., Rai S.K., Tiwari M., Chandra R. (2007). Effect of hyperhomcysteinemia on cardiovascular risk factors and initiation of atherosclerosis in Wistar rats. Eur. J. Pharamcol..

[305] Zou C.-G., Banerjee R. (2005). Homocysteine and redox signaling. Antioxid. Redox Signal..

[306] Banerjee R., Zou C.-G. (2005). Redox regulation and reaction mechanism of human cystathionine-beta-synthase: A PLP-dependent hemesensor protein. Arch. Biochem. Biophys..

[307] James S.J., Cutler P., Melnyk S. (2004). Metabolic biomarkers of increased oxidative stress and impaired methylation capacity in children with autism. Am. J. Clin. Nutr..

[308] Prudova A., Bauman Z., Braun A. (2006). S-adenosylmethionine stabilizes cystathionine beta-synthase and modulates redox capacity. Proc. Natl. Acad. Sci. USA.

[309] Reis E.A., Zugno A.I., Franzon R. (2002). Pretreatment with vitamins E and C prevent the impairment of memory caused by homocysteine administration in rats. Metab. Brain Dis..

[310] Murr C., Widner B., Wirleeitner B., Fuchs D. (2001). Neopterin as a marker for immune system activation. Curr. Drug Metab..

[311] Aykutoglu G., Tartik M., Darendelioglu E., Ayna A., Baydas G. (2020). Melatonin and vitamin E alleviate homocysteine-induced oxidative injury and apoptosis in endothelial cells. Mol. Biol. Rep..

[312] Kumar D., Jugdutt B.I. (2003). Apoptosis, and oxidants in the heart. J. Lab. Clin. Med..

[313] Tartik M., Darendelioglu E., Aykutoglu G., Baydas G. (2016). Turkish propolis supresses MCF-7 cell death induced by homocysteine. Biomed. Pharmacother..

[314] Cordaro M., Siracusa R., Fusco R., Cuzzocrea S., Di Paola R., Impellizzeri D. (2021). Involvements of Hyperhomocysteinemia in Neurological Disorders. Metabolites.

[315] Toya T., Sara J.D., Lerman B., Ahmad A., Taher R., Godo S., Corban M.T., Lerman L.O., Lerman A. (2020). Elevated plasma homocysteine levels are associated with impaired peripheral microvascular vasomotor response. Int. J. Cardiol. Heart Vasc..

[316] Ahmad A., Corban M.T., Toya T., Sara J.D., Lerman B., Park J.Y., Lerman L.O., Lerman A. (2020). Coronary Microvascular Endothelial Dysfunction in Patients With Angina and Nonobstructive Coronary Artery Disease Is Associated With Elevated Serum Homocysteine Levels. J. Am. Heart Assoc..

[317] Ji Y., Li X., Teng Z., Li X., Jin W., Lv P.Y. (2020). Homocysteine is Associated with the Development of Cerebral Small Vessel Disease: Retrospective Analyses from Neuroimaging and Cognitive Outcomes. J. Stroke Cerebrovasc. Dis..

[318] Park S., Park G.M., Ha J., Cho Y.R., Roh J.H., Park E.J., Yang Y., Won K.B., Ann S.H., Kim Y.G. (2020). Homocysteine is not a risk factor for subclinical coronary atherosclerosis in asymptomatic individuals. PLoS ONE.

